# Identification of Circular RNA-Based Immunomodulatory Networks in Colorectal Cancer

**DOI:** 10.3389/fonc.2021.779706

**Published:** 2022-01-27

**Authors:** Zongfeng Feng, Leyan Li, Yi Tu, Xufeng Shu, Yang Zhang, Qingwen Zeng, Lianghua Luo, Ahao Wu, Wenzheng Chen, Yi Cao, Zhengrong Li

**Affiliations:** ^1^ Department of General Surgery, First Affiliated Hospital of Nanchang University, Nanchang, China; ^2^ Laboratory of Digestive Surgery, Nanchang University, Nanchang, China; ^3^ Medical Innovation Center, the First Affiliated Hospital of Nanchang University, Nanchang, China; ^4^ Queen Mary School, Medical Department of Nanchang University, Nanchang, China; ^5^ Department of Pathology, the First Affiliated Hospital of Nanchang University, Nanchang, China

**Keywords:** circRNA, RPPH1, ceRNA network, RBP, M2 macrophages, Immunomodulatory

## Abstract

**Background:**

Circular RNAs (circRNAs) have been recently proposed as hub molecules in various diseases, especially in tumours. We found that circRNAs derived from ribonuclease P RNA component H1 (RPPH1) were highly expressed in colorectal cancer (CRC) samples from Gene Expression Omnibus (GEO) datasets.

**Objective:**

We sought to identify new circRNAs derived from RPPH1 and investigate their regulation of the competing endogenous RNA (ceRNA) and RNA binding protein (RBP) networks of CRC immune infiltration.

**Methods:**

The circRNA expression profiles miRNA and mRNA data were extracted from the GEO and The Cancer Genome Atlas (TCGA) datasets, respectively. The differentially expressed (DE) RNAs were identified using R software and online server tools, and the circRNA–miRNA–mRNA and circRNA–protein networks were constructed using Cytoscape. The relationship between targeted genes and immune infiltration was identified using the GEPIA2 and TIMER2 online server tools.

**Results:**

A ceRNA network, including eight circRNAs, five miRNAs, and six mRNAs, was revealed. Moreover, a circRNA–protein network, including eight circRNAs and 49 proteins, was established. The targeted genes, ENOX1, NCAM1, SAMD4A, and ZC3H10, are closely related to CRC tumour-infiltrating macrophages.

**Conclusions:**

We analysed the characteristics of circRNA from RPPH1 as competing for endogenous RNA binding miRNA or protein in CRC macrophage infiltration. The results point towards the development of a new diagnostic and therapeutic paradigm for CRC.

## Introduction

Colorectal cancer (CRC) is the most common malignant tumour of the digestive tract. CRC cases diagnosed each year exceed 1.9 million globally and account for 935,000 cancer-related deaths annually (1). Patients with advanced CRC have few treatment options, poor prognosis, and high mortality rates. Chemotherapy, targeted therapy, and immunotherapy are the most commonly used methods to treat advanced CRC. However, patients are prone to drug resistance, which often leads to treatment failure (2). Therefore, it is vital to elucidate the pathogenesis of CRC to develop and utilise new drugs.

Circular RNAs (circRNAs) are circular transcripts that are more stable than their linear counterparts; they lack the termini by which certain RNAses bind, conferring them resistance to degradation by pH and enzymatically catalysed hydrolysis. CircRNA can adsorb miRNAs (3) and proteins (4), and, intriguingly, some circRNAs even have translation functions (5). Emerging research has shown circRNAs’ important role in various biological processes in tumour cells, especially in colon cancer. Peng et al. (6) suggested that circCUL2 mediates the miR-142-3p/ROCK2 axis to induce autophagy activation and regulate cisplatin sensitivity. Meanwhile, circPTK2 is well expressed in CRC tissue and cells and is associated with metastasis, making it a novel therapeutic target for metastatic colorectal cancer (7). Although studies have clarified the involvement of circRNAs in the biological processes in tumour cells, the role of circRNAs in immunoregulation remains unclear.

Macrophages take on two opposing roles: M1 macrophages are pro-inflammatory, whereas M2 macrophages are anti-inflammatory. M1 cells can swallow atypical or tumour cells and have a positive effect on the resilience of the immune system to tumour infiltration. Conversely, after induced polarisation to the M2 form, macrophages can help tumour cells to escape immune surveillance, promoting the proliferation and metastasis of tumour cells (8). Several studies have shown that M2 and tumour-associated macrophages (TAMs) are related to the poor prognosis of colon cancer patients and may promote tumour progression and metastasis (9, 10). Details of the polarisation process of macrophages and the mechanisms by which they aid tumour cells in immune system evasion remain undetermined.

Ribonuclease P NRA component H1 (RPPH1), a non-coding RNA (ncRNA) found on chromosome 14, plays a key role in several human tumours. Wu et al. (11) demonstrated that RPPH1 promotes non-small-cell lung cancer progression and resistance to standard cis-platinum drugs by targeting the WNT2B signalling axis miR-326. RPPH1 expression was also suggested to be associated with prognosis and further interfered with tumour suppressor factor p21 in gastric cancer (12). Moreover, Liang et al. (13) demonstrated that RPPH1 overexpression mediates macrophage M2 polarisation to promote colorectal cancer metastasis. CD44v6, the sixth variant exon of the CD44 gene, has been reported to contribute to CRC progression (14, 15). NCAM1, a surface biomarker of natural killer (NK) cells, is also known as CD56 (16). Gharagozloo (17) reported that patients with metastatic colorectal cancer had significantly lower CD56+ NKT cell counts in the peripheral blood than did healthy individuals.

Here, we aimed to further investigate the role of RPPH1 in the molecular background of CRC by identifying circRNAs deriving from this gene and assessing the association between macrophages and targeted genes. In total, eight circRNAs derived from RPPH1 were identified; their expression in CRC cells was assessed, and bioinformatics tools were used to predict their interactions.

## Methods

### Datasets of CRC

The circRNA expression profiles (GSE126094) (18) were downloaded from the Gene Expression Omnibus (GEO) database (https://www.ncbi.nlm.nih.gov/geo/). The miRNA and mRNA expression data for CRC were obtained from The Cancer Genome Atlas (TCGA) database (https://portal.gdc.cancer.gov/). The colon cancer mutation data were also downloaded from TCGA database.

### Differentially Expressed circRNAs, miRNAs, and mRNAs

The differentially expressed (DE) circRNAs and DEmRNAs were screened in CRC patients and healthy individuals using the Bioconductor Limma package (version 3.48.1) (19). The thresholds of adjusted p < 0.05 and |log FC > 1| were used to identify DEcircRNA from GSE126094 and DEmRNA from TCGA colorectal cancer datasets. The starBase v2.0 online web server (http://starbase.sysu.edu.cn/) (20) was used to analyse DEmiRNA from TCGA colon cancer datasets.

### Establishment of circRNA–miRNA–mRNA Network

CircRNA was visualised using the CSCD2.0 database (http://gb.whu.edu.cn/CSCD2/#). We first predicted the target-absorbed miRNAs of eight circRNAs using the circBank database (21); we then verified these prediction results and visualised the binding sites of miRNA adsorption using the circMIR1.0 software. We screened miRNAs with expression differences in the TCGA colon cancer datasets and found those that overlapped with the targeted miRNA. Next, the miRNA-forecasted mRNA and overlapping portions between the DEmRNAs were chosen to establish an miRNA–mRNA network related to CRC tumorigenesis. The MiRWalk (22) database, a comprehensive online target predition tool, was used for the prediction of miRNA-targeted mRNA. To ensure more reliable results, a score of >0.95 for miRNA–mRNA pairs present in both the miRDB and miRTarbase miRNA-target prediction datasets were chosen for further study. Finally, we used Cytoscape software (version 3.6.0) (23) to visualise the competing endogenous RNA (ceRNA) network.

### Construction of Protein–Protein Interaction Network and Identification of the Hub Genes

We constructed a protein–protein interaction (PPI) network *via* the STRING database (https://string-db.org/) (24) based on the DEmRNA in the ceRNA network. We set a score of >0.4 to filter the criterion in this PPI network. The intersections between the adjacent nodes determined to be ≥5 using R software were regarded as hub genes. Subsequently, we established a secondary circRNA–miRNA–mRNA subnetwork according to the identified hub genes.

### Survival and Drug Sensitivity Analysis of the Hub Genes

To cross-validate the reliability of hub genes, the Xiantao search tool (https://www.xiantao.love/) was used to compare six hub genes by difference analysis and paired difference analysis derived from the TCGA database. The PrognoScan (http://www.prognoscan.org/) database was used to analyse the overall survival (OS) derived from the GEO database. The correspondence between hub genes and sensitivity to drugs was explored using the online search tool GSCALite (25).

### Construction of circRNA–Protein Regulatory Network

We used the RBPmap tool (http://rbpmap.technion.ac.il/) to study the interaction between these circRNAs and RBPs (26). This tool takes custom RNA sequences as input and provides a list of RBP binding sites and assigns them probability values.

### Relationship Between Targeted Genes and Immune Cells

We used the GEPIA2 server (http://gepia2.cancer-pku.cn/) to analyse the relationship between the expression of the four genes and the state of immune cell infiltration. Then, the XCELL, TIMER, CIBERSORT, CIBERSORT-ABS, QUANTISEQ, MCP-COUNTER, and EPIC algorithms were used to further evaluate the macrophage immune infiltration of CRC with TIMER2 (http://timer.cistrome.org/) (27).

### Cell Culture and qRT-PCR Assays

Six CRC cell lines, namely, Caco-2, HCT-116, HT-29, SW-480, SW-620, and DLD-1, and normal intestinal mucosal epithelial cells (NCM-460) were cultured in Roswell Park Memorial Institute (RPMI) 1640 medium with 10% fetal bovine serum. Total RNA was extracted from CRC cell lines and reverse transcribed into cDNA with random primers, following the manufacturers’ instructions. The amplified region of the primer design included the circRNA looped linker region. The main benefit of this approach is that it ensures the specificity of the primer. Glyceraldehyde 3-phosphate dehydrogenase (GAPDH) expression was used as an internal reference to normalise circRNAs and mRNA, and U6 expression was used as internal reference for miRNAs. **Table 1** and **Supplementary Table S1** described the circRNA, miRNA, and mRNA primers. Excel software was used for PCR data analysis, and GraphPad was used for mapping. First, the quantification cycle (Cq) mean of the internal reference gene in the sample was calculated, and then, the Cq difference (ΔCq) between the target gene and internal reference gene was determined. Furthermore, the 2^^−ΔΔCq^ formula was used to calculate the expression level. Finally, based on the expression level of NCM-460 cells, the relative expression level of the target genes in other colon cancer cell lines were calculated.

**Table 1:** Primers for circular RNA (circRNA) amplification.

Primer name	Sequence (5′ to 3′)
hsa_circ_0000511-F	TTTGCCGGAGCTTGGAACA
hsa_circ_0000511-R	CGTTCTCTGGGAACTCACCTC
hsa_circ_0000512-F	AGTTCAATGGCTGAGGTGAGG
hsa_circ_0000512-R	TGTTCCAAGCTCCGGCAAAG
hsa_circ_0000514-F	GGTCAGACTGGGCAGGAGAT
hsa_circ_0000514-R	CCCGTTCTCTGGGAACTCAC
hsa_circ_0000515-F	GGTCAGACTGGGCAGGAGAT
hsa_circ_0000515-R	GAGTGACAGGACGCACTCAG
hsa_circ_0000517-F	GGGAGGTGAGTTCCCAGAG
hsa_circ_0000517-R	CAGGGAGAGCCCTGTTAGG
hsa_circ_0000518-F	GTGAGTTCCCAGAGAACGGG
hsa_circ_0000518-R	GAGTGACAGGACGCACTCAG
hsa_circ_0000519-F	CTAACAGGGCTCTCCCTGAG
hsa_circ_0000519-R	CAGACCTTCCCAAGGGACAT
hsa_circ_0000520-F	GGGAAGGTCTGAGACTAGGG
hsa_circ_0000520-R	GGACATGGGAGTGGAGTGAC

## Results

### Differentially Expressed circRNAs, miRNAs, and mRNAs in Colon Cancer

**Figure 1:**
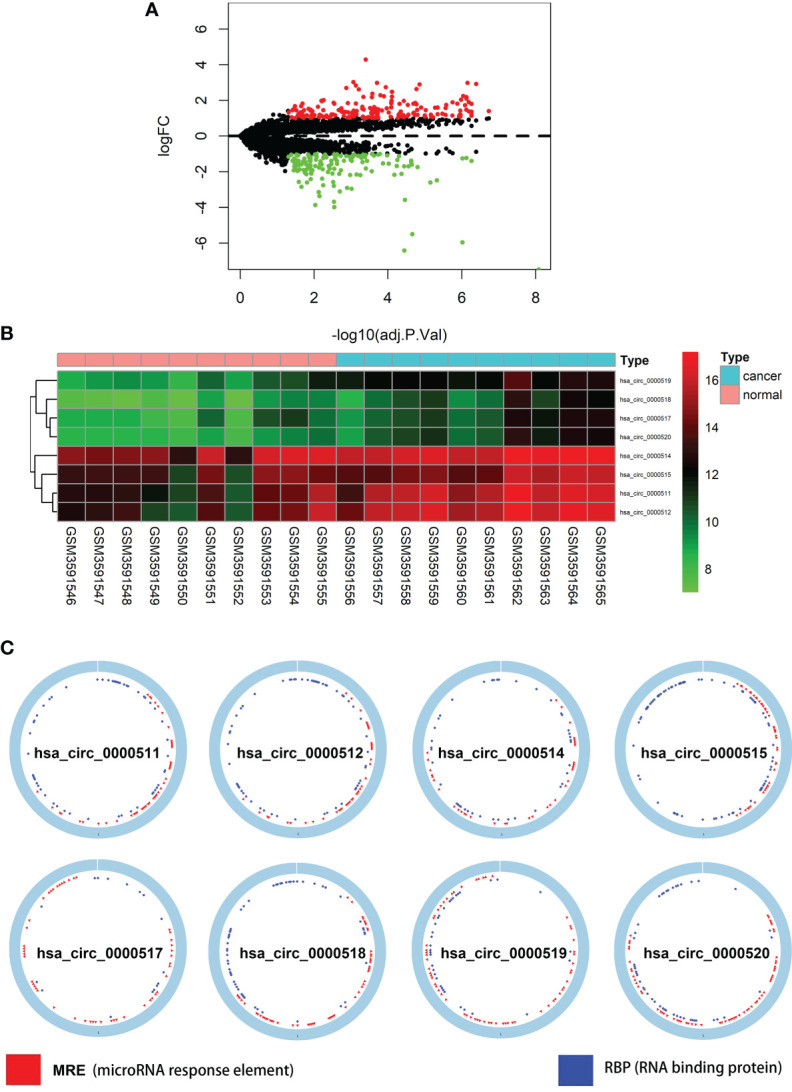
Differentially expressed circular RNAs (circRNAs) from RPPH1 in colorectal cancer (CRC). **(A)** Volcano plots for differentially expressed circRNAs (DEcircRNAs) in CRC from the GSE126094 dataset. **(B)** Heatmap of the eight differentially expressed circRNAs from the RPPH1 gene. **(C)** Interaction patterns of the eight circRNAs based on CSCD2.

**Figure 2:**
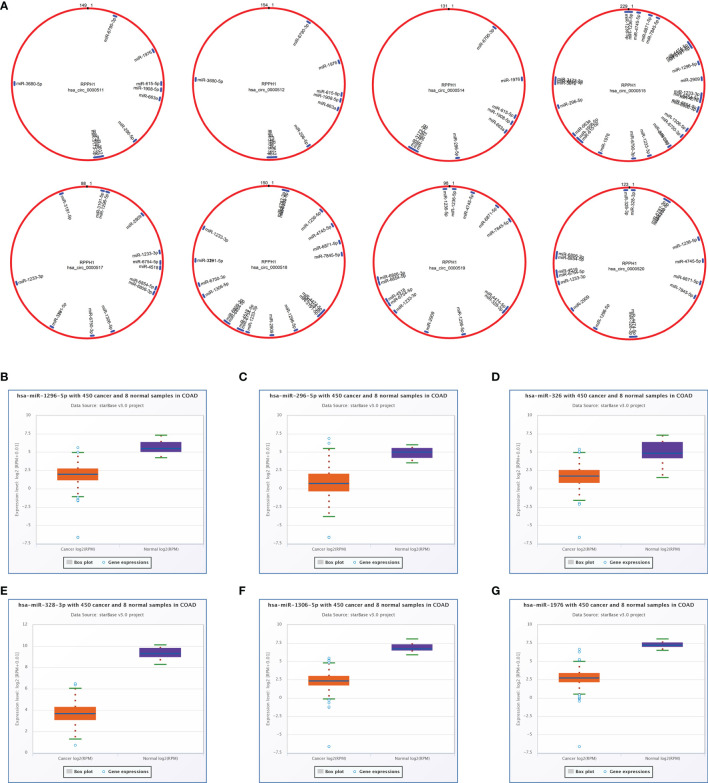
Targeted sponge miRNA with eight circular RNAs (circRNAs). **(A)** The position and quantity of miRNA adsorption *via* circMIR1.0 software. **(B–G)** Six targeted sponge miRNAs with different expression levels in colorectal cancer (CRC) from starBase v2.0. **(B)** hsa-miR-1296-5p, **(C)** hsa-miR-296-5p, **(D)** hsa-miR-326, **(E)** hsa-miR-328-3p, **(F)** hsa-miR-1306-5p, **(G)** hsa-miR-1976.

The total number of 321 DEcircRNAs (179 upexpressed and 142 downexpressed) in GSE126094 was identified (**Figure 1A**). Eight co-upregulated circRNAs (hsa_circ_0000511, hsa_circ_0000512, hsa_circ_0000514, hsa_circ_0000515, hsa_circ_0000517, hsa_circ_0000518, hsa_circ_0000519, and hsa_circ_0000520) originating from the RPPH1 gene were identified (**Figure 1B**). **Figure 1C** shows eight circRNA-binding sites that potentially competitively bind to miRNA and RBPs. circMIR1.0 predicted the miRNAs bound by circRNAs and the corresponding binding regions (**Figure 2A**). Since eight circRNAs had the same nucleic acid sequences in multiple regions, their predicted miRNAs were mostly similar. Six downregulated miRNAs were identified in TCGA colon datasets containing 450 colon cancer and eight normal samples (hsa-miR-1296-5p, hsa-miR-296-5p, hsa-miR-326, hsa-miR-328-3p, hsa-miR-1306-5p, and hsa-miR-1976) (**Figures 2B–G**). A total of 7,877 DEmRNAs (5,590 upexpressed and 2,287 downexpressed) were authenticated from the TCGA colon cancer datasets.

### CircRNA–miRNA–mRNA and PPI Networks

**Figure 3:**
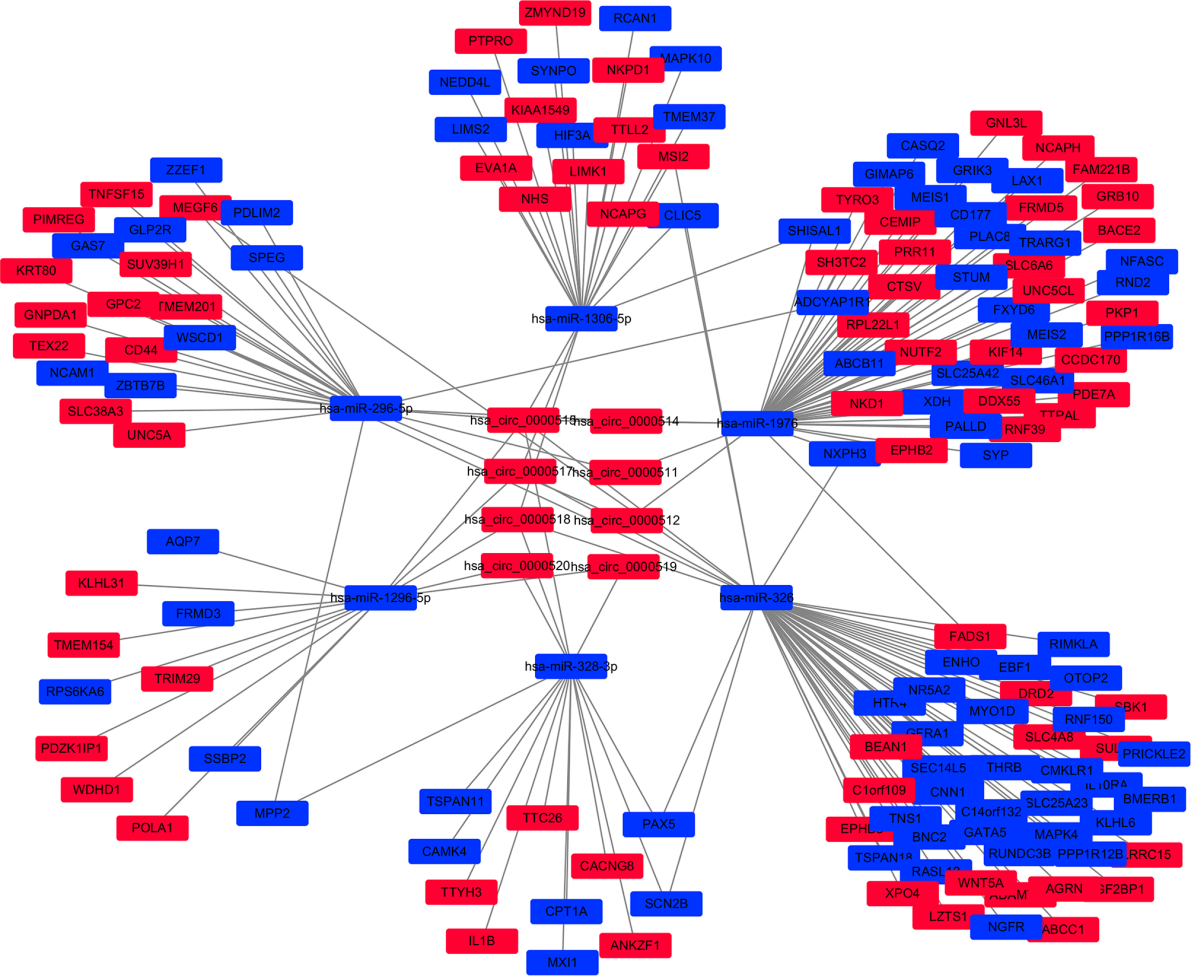
The circular RNA (circRNA)–miRNA–mRNA network in the colorectal cancer (CRC) by Cytoscape v3.6.0. The red and blue nodes represent upregulation and downregulation, respectively.

**Figure 4:**
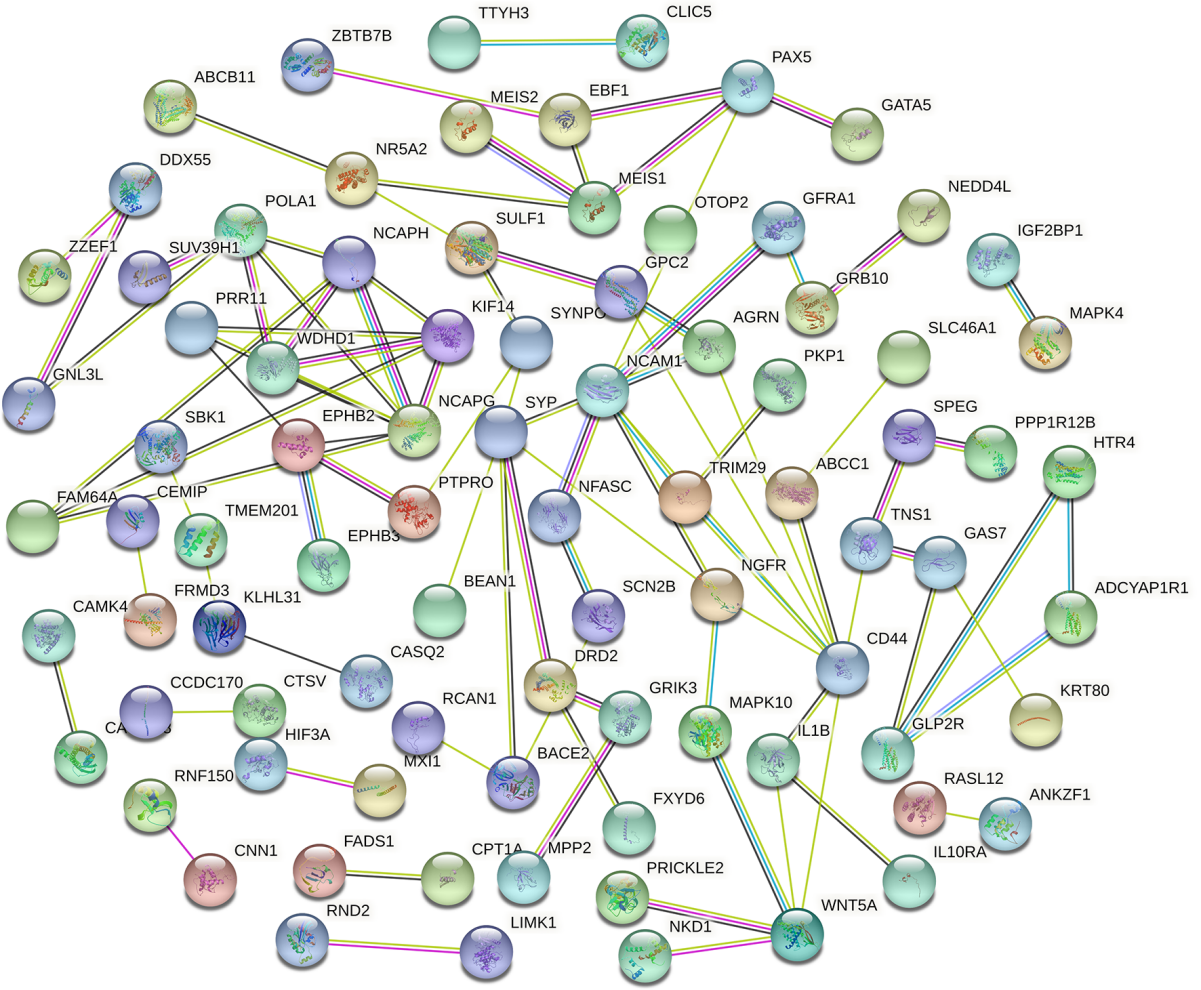
Protein–protein intersection (PPI) network analysis of differentially expressed mRNA (DEmRNA) involved in the competing endogenous RNA (ceRNA) network.

Cytoscape v3.6.0 was used to analyse and establish a circRNA–miRNA–mRNA network based on regulated and differentially expressed ceRNAs. The network included 8 circRNAs, 6 miRNAs, and 162 mRNAs (**Figure 3**). As described in **Figure 4**, to show the interaction of target genes in CRC, we constructed a PPI network based on the STRING database to include 151 differentially expressed genes predicted to be regulated by miRNAs.

### Hub Gene and ceRNA Subnetwork

**Figure 5:**
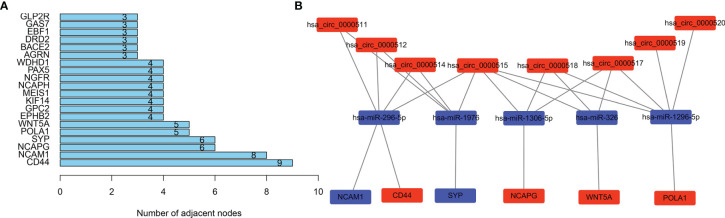
Selecting hub genes and constructing circular RNA (circRNA)–miRNA–hub gene subnetwork. **(A)** Selecting hub genes: WNT5A, POLA1, SYP, NCAPG, NCAM1, and CD44 by analysing the protein–protein interaction (PPI) network. Adjacent differential gene interaction nodes are ≥5. **(B)** Reconstructing a circRNA–miRNA–hub gene subnetwork based on the circRNA–miRNA–mRNA network and hub genes.

Genes from the PPI network in the STRING database, with adjacent differential gene interaction nodes ≥ 5 were regarded as hub genes potentially related to CRC: WNT5A, POLA1, SYP, NCAPG, NCAM1, and CD44 (**Figure 5A**). The ceRNA subnetwork, containing eight circRNAs, five miRNAs, and six mRNAs, as was established, is indicated in **Figure 5B** (hsa_circ_0000511\hsa-miR-296-5p\NCAM1, hsa_circ_0000511\hsa-miR-296-5p\CD44, hsa_circ_0000511\hsa-miR-1976\SYP, hsa_circ_0000512\hsa-miR-296-5p\NCAM1, hsa_circ_0000512\hsa-miR-296-5p\CD44, hsa_circ_0000512\hsa-miR-1976\SYP, hsa_circ_0000514\hsa-miR-296-5p\NCAM1, hsa_circ_0000514\hsa-miR-296-5p\CD44, hsa_circ_0000514\hsa-miR-1976\SYP, hsa_circ_0000515\hsa-miR-296-5p\NCAM1, hsa_circ_0000515\hsa-miR-296-5p\CD44, hsa_circ_0000515\hsa-miR-1976\SYP, hsa_circ_0000515\hsa-miR-1306-5p\NCAPG, hsa_circ_0000515\hsa-miR-326\WNT5A, hsa_circ_0000515\hsa-miR-1296-5p\POLA1, hsa_circ_0000517\hsa-miR-1306-5p\NCAPG, hsa_circ_0000517\hsa-miR-326\WNT5A, hsa_circ_0000517\hsa-miR-1296-5p\POLA1, hsa_circ_0000518\hsa-miR-1306-5p\NCAPG, hsa_circ_0000518\hsa-miR-326\WNT5A, hsa_circ_0000518\hsa-miR-1296-5p\POLA1, hsa_circ_0000519\hsa-miR-1296-5p\POLA1, and hsa_circ_0000520\hsa-miR-1296-5p\POLA1).

### Survival Analysis and Resistance of the Hub Genes

**Figure 6:**
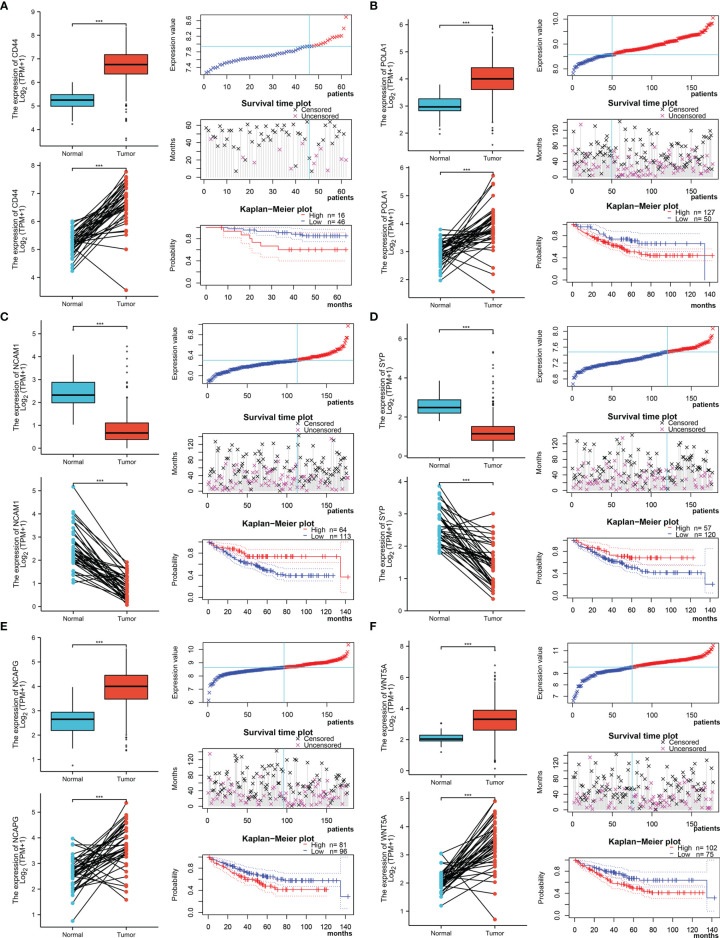
Different expression and survival analysis of the hub genes by the Xiantao and PrognoScan search tools. **(A)** For CD44, **(B)** for POLA1, **(C)** for NCAM1, **(D)** for SYP, **(E)** for NCAPG, and **(F)** for WNT5A. (***p ≤ 0.001).

**Figure 7:**
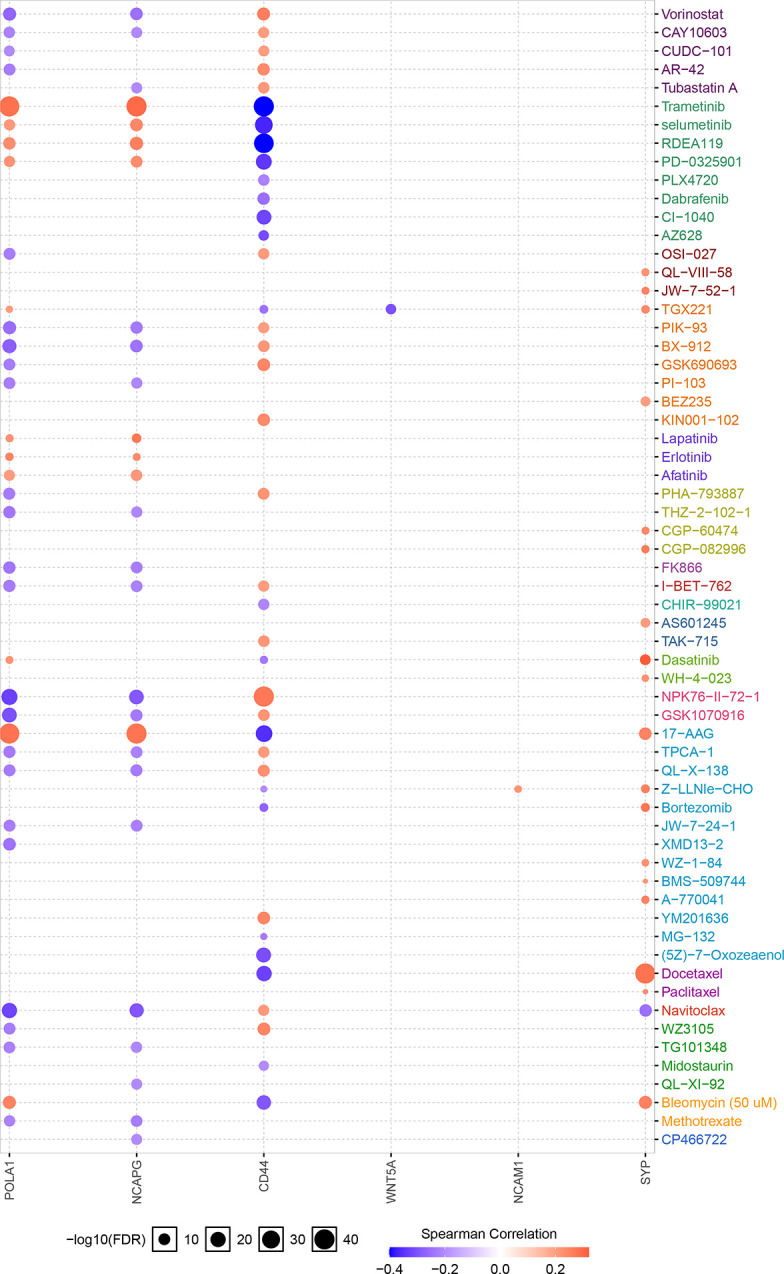
Correlation between hub gene expression levels and small molecule/drug sensitivity *via* the online search tool GSCALite.

The Xiantao search tool was used to compare six hub genes by difference analysis and paired difference analysis derived from the TCGA database, and PrognoScan was used to analyse the overall survival (OS) derived from the GEO database. As shown in **Figure 6A**, CD44 expression was significantly higher in CRC patients than in the control (healthy individals). CD44-positive patients had a shorter survival than did CD-negtive patients in the GSE12945 dataset. The hub genes POLA1, NCAPG, and WNT5A were well expressed in CRC patients and had similar prognosis results in the GSE17536 dataset (**Figures 6B, E, F**). In contrast, **Figures 6C, D** show that the hub genes NCAM1 and SYP were expressed at lower levels in colon cancer patients than in the control. From the GSE17536 dataset, patients with lower expression levels of POLA1, NCAPG, and WNT5A had better survival than those with higher expression levels. In some instances, the expression levels of NCAM1 and SYP inhibited CRC progression. Subsequently, we performed a drug sensitivity (IC50) evaluation of the expression levels of the targeted-network genes and found that a higher correlation represented a higher drug resistance. The expression levels of POLA1 and NCAPG were positively correlated with sensitivity to trametinib and the 17-AAG (HSP90 inhibitor). CD44 expression was positively correlated with sensitivity to the NPK76-II-72-1 (kinase inhibitor). Moreover, SYP expression was positively correlated with sensitivity to docetaxel (**Figure 7**). Therefore, drug sensitivity analysis could help toward the individualized and precise treatment of tumour patients, reducing the occurrence of tumour drug resistance.

### Copy Number Variation and Mutation of Hub Genes

**Figure 8:**
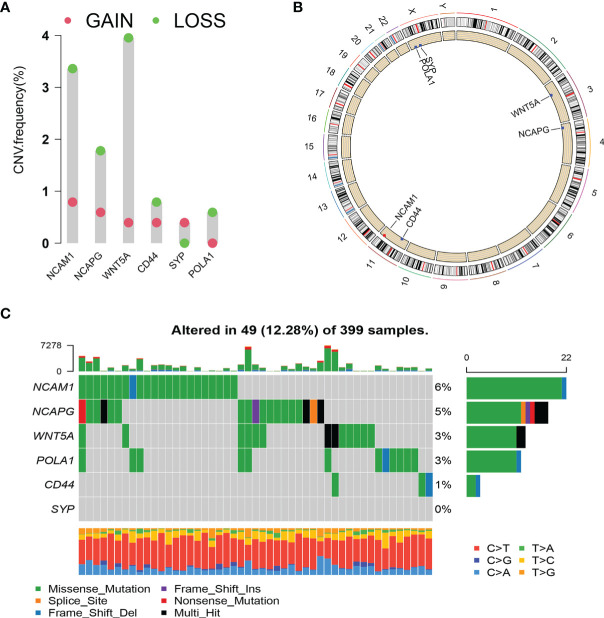
Copy number variation (CNV) and mutation of hub genes. **(A)** CNV frequency of hub genes. **(B)** Circle diagram of CNV with hub genes. Red represents CNV gain; blue represents CNV loss. **(C)** Cascade of core gene mutations.

We analysed the copy number variation (CNV) and gene mutation of hub genes in TCGA colon cancer data. In CRC samples, the CNV mutation frequency of WNT5A reached 4%, mainly with CNV deletion. This was followed by NCAM1, while the CNV mutation frequency in CD44, SYP, and POLA1 was <1% (**Figure 8A**). **Figure 8B** shows the types of CNV mutations and chromosomal statuses of the hub genes. Among the 399 samples expressing the six hub genes, 49 (12.28%) were found to have gene mutations, most of which were missense mutations. NCAM1 accounted for 6%, NCAPG accounted for 5%, WNT5A and POLA1 both accounted for 3%, and CD44 accounted for 1% (**Figure 8C**).

### The CircRNA-Binding Protein Network

**Figure 9:**
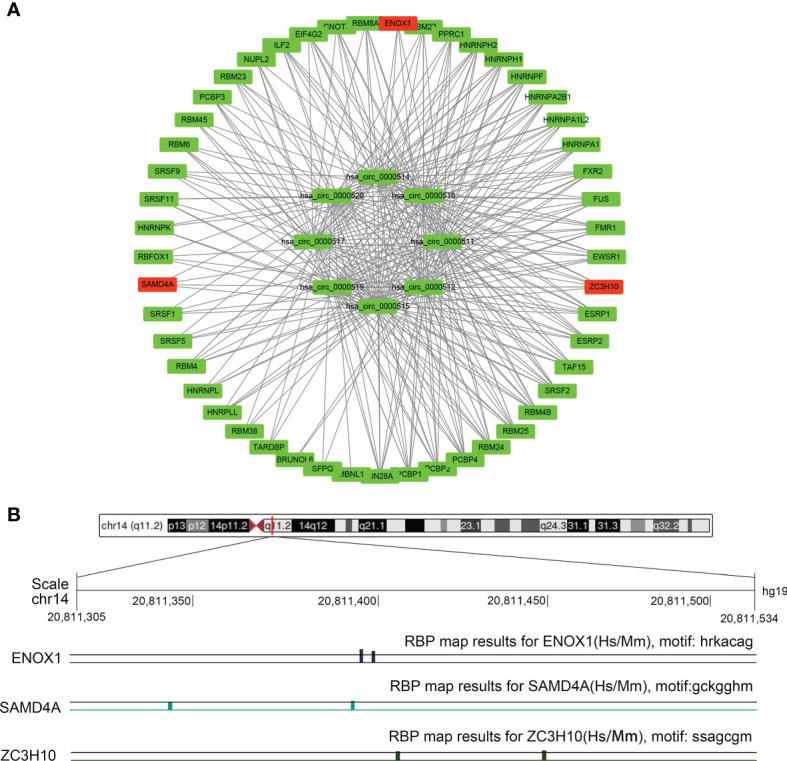
The circRNA–protein network and binding site in the CRC. **(A)** Circular RNA (circRNA)–protein network in colorectal cancer (CRC) according to the RBPmap. The red nodes represent immune-related proteins, whereas the green nodes represent other proteins and eight circRNAs. **(B)** Chromosome positions and ENOX1, NCAM1, SAMD4A, and ZC3H10 binding sites of hsa_circ_0000515.

As depicted in **Figure 1C** for eight circRNAs, various protein-binding sites were detected, and many RBPs were predicted to bind to them. The RBPmap provided more information about the number of RBPs and their binding sites. We selected RBPs with p < 0.05, accompanied by high probability values (**Figure 9A**; **Supplementary Table S2**). Since these circRNAs all originate from the RPPH1 gene, their RNA-binding proteins are all the same, although some specific regions have different sequences and RBPs. Further analysis revealed that ZC3H10, SAMD4A, and ENOX1 are closely related to anti-tumour immunity against colon cancer. **Figure 9B** shows the number of sites and positions where has_circ_0000515 binds to ZC3H10, SAMD4A, and ENOX1.

### Relationship Between Immune-Related Genes and Macrophages

We explored the possible molecular mechanisms underlying the aetiology of CRC through immune cell infiltration. In particular, the relationship between four target genes, namely, NCAM1, ZC3H10, SAMD4A, and ENOX1, and macrophages was investigated.

**Figure 10:**
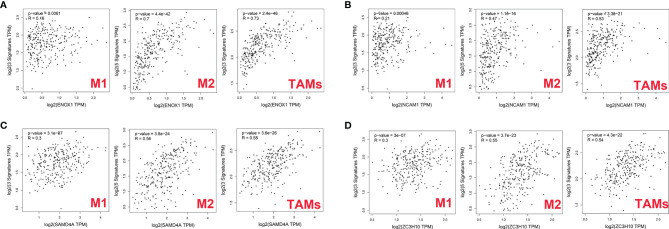
Correlation between targeted gene expression levels and markers of macrophages as analysed *via* GEPIA2. **(A)** for ENOX1, **(B)** for NCAM1, **(C)** for SAMD4A, and **(D)** for ZC3H10.

First, we used GEPIA2 to analyse the association between the expression levels of the four genes and M1 macrophage (NOS2, IRF5, and PTGS2), M2 macrophage (MS4A4A, VSIG4, MRC1, CD163, and MSR1), and TAM (CCL2, CD68, and IL10) markers. There was a positive correlation between ENOX1 expression and the M2 macrophage (R = 0.7, *p* = 4.4E−42), and TAM (R = 0.73, *p* = 2.4E−46), whereas the correlation with M1 macrophage marker genes (R = 0.16, *p* = 0.0061) was poor (**Figure 10A**). **Figure 10B** shows the correlation between NCAM1 and M2 macrophage (R = 0.47, *p* = 1.1E−16), TAM (R = 0.53, *p* = 3.3E−21), and M1 macrophage (R = 0.21, *p* = 0.00046). **Figure 10C** shows the correlation between SAMD4A and M2 macrophage (R = 0.56, *p* = 3.8E−24), TAM (R = 0.58, *p* = 3.6E−26), and M1 macrophage (R = 0.30, *p* = 3.1E−07). **Figure 10D** shows the correlation between ZC3H10 and M2 macrophage (R = 0.55, *p* = 3.7E−23), TAM (R = 0.54, *p* = 4.3E−22), and M1 macrophage (R = 0.30, *p* = 3.1E−07). These results strongly suggest that these genes are closely related to the polarisation of macrophages in colon cancer development. We observed similar results between ENOX1 expression and other different types of immune cells, such as CD4+ cells (R = 0.70, *p* = 2.4E−42) and NK cells (R = 0.72, *p* = 3.5E−45) (**Supplementary Figure S1**). Similar to ENOX1, the expression levels of NCAM1, SAMD4A, and ZC3H10 were positively correlated with the marker expression levels of immune cells (**Supplementary Figures S2**-**4**), indicating that the expression levels of these genes are closely related to tumour immunity.

**Figure 11:**
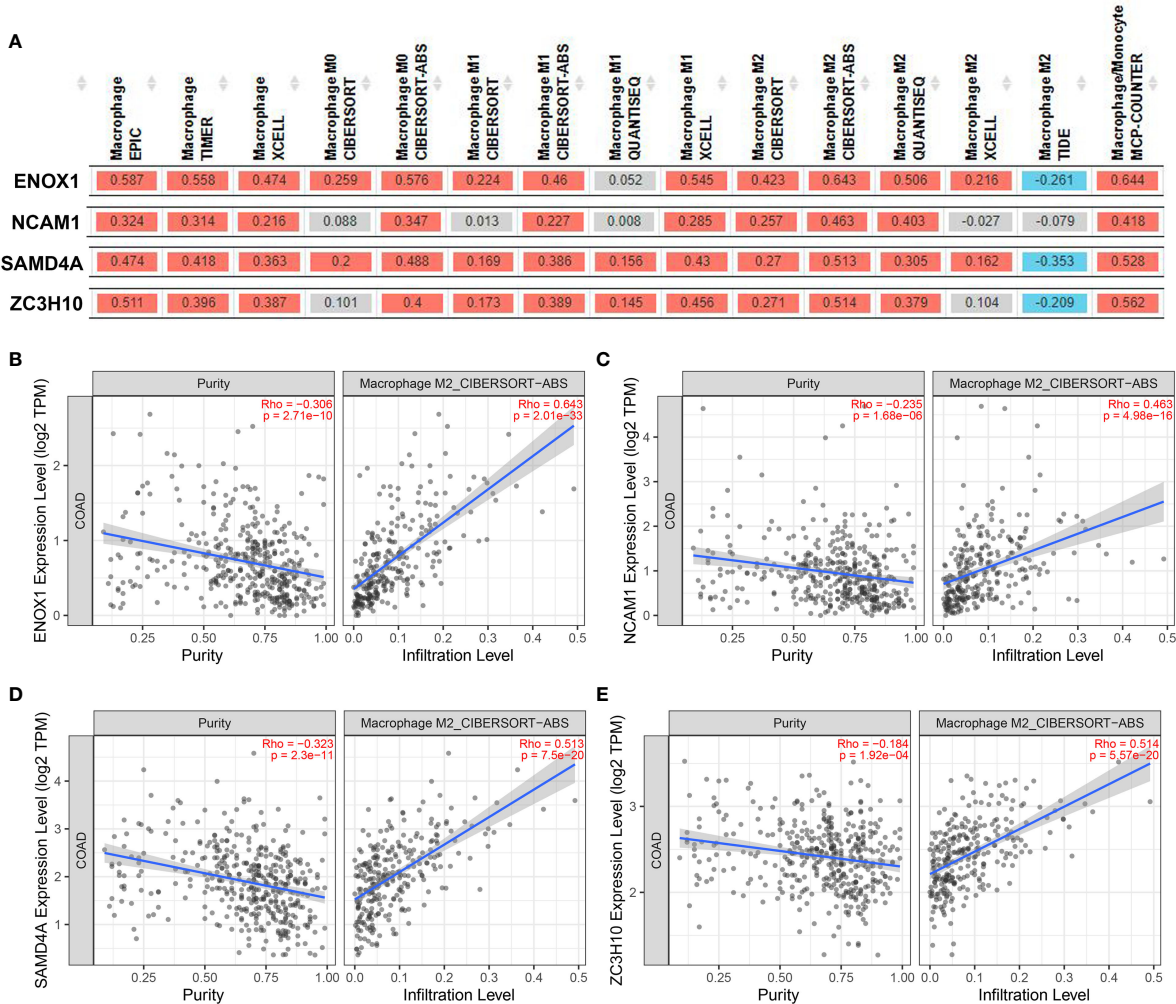
Correlation between targeted gene expression levels and the infiltration level of macrophage cells. The XCELL, TIMER, CIBERSORT, CIBERSORT-ABS, QUANTISEQ, MCP-COUNTER, and EPIC algorithms were applied for immune infiltration estimations with ENOX1, NCAM1, SAMD4A, and ZC3H10. **(A)** Overview of the correlations between ENOX1, NCAM1, SAMD4A, and ZC3H10 and the macrophages. The CIBERSORT-ABS algorithm was used to estimate the correlations between ENOX1, NCAM1, SAMD4A, and ZC3H10 and the M2 macrophages after tumour purity adjusting in panels **(B–E)**. **(B)** ENOX1 and M2 macrophages, **(C)** NCAM1 and M2 macrophages, **(D)** SAMD4A and M2 macrophages, **(E)** ZC3H10 and M2 macrophages.

We then used the XCELL, TIMER, CIBERSORT, CIBERSORT-ABS, QUANTISEQ, MCP-COUNTER, and EPIC algorithms to further evaluate the macrophage immune infiltration of CRC. All algorithms except XCELL found positive correlations between these four immune-related genes and M2 macrophages that were significantly higher than those with M1 macrophages (**Figure 11A**). Significant correlation was observed between the polarised M2 macrophages and ENOX1, NCAM1, SAMD4A, and ZC3H10 with respect to tumour purity (**Figures 11B–E**). The consistency of **Figures 10** and **11** further confirm the reliability of the relationship between these expression levels of these genes and polarisation of macrophages.

### Expression Analysis of RPPH1 circRNAs, miRNA, and mRNA in CRC Cells

**Figure 12:**
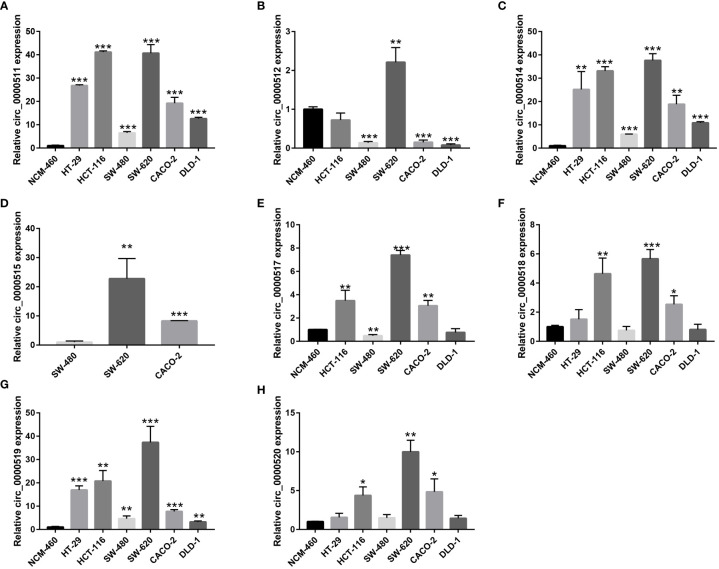
Relative circular RNA (circRNA) expression in colorectal cancer (CRC) cells and NCM-460 normal cells by qRT-PCR. **(A)** For hsa_circ_0000511, **(B)** for hsa_circ_0000512, **(C)** for hsa_circ_0000514, **(D)** for hsa_circ_0000515, **(E)** for hsa_circ_0000517, **(F)** for hsa_circ_0000518, **(G)** for hsa_circ_0000519, and **(H)** for hsa_circ_0000520. (***p≤0.001, **p≤0.01, *p≤0.05).

The expression levels of the circRNAs derived from RPPH1 in CRC cells were detected using quantitative reverse transcription PCR (qRT-PCR) assays (**Supplementary Table S3**). Hsa_circ_0000511 (**Figure 12A**), hsa_circ_0000514 (**Figure 12C**), and hsa_circ_0000519 (**Figure 12G**) were more expressed in all cancer cell lines than in the NCM-460 control cells. Compared with NCM-460, hsa_circ_0000512 had the highest expression levels in SW-620 cells and low expression in SW-480, CACO-2, and DLD-1, and was not detected in HT-29 cells (**Figure 12B**). Hsa_circ_0000515 was found to be expressed in SW-480, SW-620, and CACO-2 but was not detected in other CRC cell lines (**Figure 12D**). As shown in **Figure 12E**, the expression of hsa_circ_0000517 was the highest in SW-620 cells and the lowest in SW-480 cells and DLD-1 cells. The expression levels of hsa_circ_0000518 and hsa_circ_0000520 were higher in HCT-116, SW-620, and CACO-2 than in NCM-460 (**Figures 12F, H** **)**. From the above results, the expression levels of circRNAs were low and even undetectable in CRC cell lines. In our results, circRNAs from the RPPH1 gene were the highest in SW-620 and CACO-2 and the lowest in NCM-460 and SW-480. MiR-296-5p was highly expressed in NCM-460 and CACO-2 compared with others. However, CD44, NCAM1,SYP, and NCAPG showed higher expression levels than NCM-460 in the majority of colon cancer cell lines (**Supplementary Figure S6**).

## Discussion

CRC is a complex malignant tumour of the digestive tract caused by a wide variety of gene mutations and signal pathway disorders. Research has revealed the importance of circRNAs as a new type of transcriptional gene regulation molecule in the pathogenesis of various human diseases, including malignant tumours. circRNA is a closed circular nucleotide sequence that specifically binds a variety of miRNAs and proteins. In recent years, the role of circRNA in cancer tumorigenesis has gradually been uncovered but has remained largely unknown in CRC. To explore the important role of circRNA in CRC as the starting trigger point for oncogene activation, we first screened DEcircRNA from GEO data and DEmiRNA and DEmRNA from TCGA data. After predicting the interactions between circRNA, miRNA, and proteins, and their effects on the strength of biology and molecular mechanics, a circRNA–miRNA–mRNA and circRNA–protein regulatory network was described. Then, a PPI network model was constructed, and the following six genes were identified as central genes: WNT5A, POLA1, SYP, NCAPG, NCAM1, and CD44. We performed expression verification and survival analysis on these six core genes based on TCGA and GEO data, respectively, which further enhanced the reliability of the ceRNA network. We also constructed a circRNA–protein gene subnetwork based on related RNA-binding proteins and further analysed their binding relationships. By combining the regulated genes in ceRNA and circRNA binding protein genes, we comprehensively analysed their correlation with tumour immune regulation.

An increasing number of studies have shown that the abnormal expression of circRNA is related to the pathogenesis of CRC, indicating that circRNAs are potential therapeutic targets and biomarkers. Chen et al. (28) demonstrated that circGLIS2 was higher in CRC patients than in healthy individuals, and the overexpression of circGLIS2 sponged miR-671 to activate the nuclear facor kappa B (NF-κB) signalling pathway. Yang et al. (7) highlighted that circPTK2, a novel circRNA, is elevated in CRC tissues, promotes the epithelial–mesenchymal transition of colorectal cancer cells, and serves as a potential target for late treatment and early diagnosis. However, our understanding of these RNA molecules is lacking, and further research is required to explore their relationship with CRC.

Eight circRNAs (hsa_circ_0000511, has_circ_0000512, has_circ_0000514, has_circ_0000515, has_circ_0000517, has_circ_0000518, has_circ_0000519, and has_circ_0000520) originating from the RPPH1 gene were identified in the ceRNA network. Five of these eight circRNAs were reported to be involved in the development of malignant tumours. Has_circ_0000511 is overexpressed in cervical cancer and regulates the miR-296-5p/HMGA1 signalling pathway axis to inhibit HeLa and SiHa proliferation and invasion (29). Has_circ_0000515 can sponge miR-326 to promote cervical cancer progression by releasing ELK1 transcription and regulate the miR-296-5p/CXCL10 signalling pathway axis to induce the pathogenesis of breast cancer (30). Hsa_circ_0000517 was found to be significantly upregulated in hepatocellular carcinoma (HCC), and the expression of hsa_circ_0000517 was associated with HCC progression. Hsa_circ_0000517 acts as a miRNA sponge to regulate HCC growth and metastasis through hsa_circ_0000517/miR-326/IGF1R and the SMAD6 signalling pathway axis (31–33). Another circRNA, hsa_circ_0000518, was found to be significantly elevated in breast cancer and can promote the progression, apoptosis, migration, and invasion of breast cancer cells by targeting miR-326/FGFR1 (34). Controversially, hsa_circ_0000520 has been reported in gastric, breast, and cervical cancers. Sun et al. (35) and Lv et al. (36) found that hsa_circ_0000520 was significantly downregulated in gastric cancer, and its overexpression may attenuate the PI3K-Akt signalling pathway, causing the reversal of resistance to Herceptin in gastric cancer cells.

Zang (37) and Zhou (38) simultaneously reported that hsa_circ_0000520 was highly expressed in breast cancer, which suggested the regulatory mechanism of hsa_circ_0000520/miR-1296. Zheng et al. (39) reported that silencing hsa_circ_0000520 blocks cell cycle progression and promotes apoptosis *via* the miR-1296/CDK2 signalling pathway axis. In contrast, Zhang et al. (40) reported that hsa_circ_0000520 overexpression decreased PAX5 expression by sponging miR-146b-3p and repressing cervical cancer cell proliferation. Our results also confirmed that these circRNAs are highly expressed in colon cancer tissues and cell lines. Nevertheless, whether they play a key role in the pathogenesis of colon cancer remains an open question requiring further exploration.

To elucidate the molecular mechanism of action of circRPPH1, circRNA–miRNA–mRNA and circRNA–protein networks were constructed. The six hub genes and four immune-related genes have been partially reported previously. The interaction between the proteins CD44 and MUC5AC conferred colon cancer cell resistance to 5-FU *via* the downregulation of p53 and p21 (41). Increasing numbers of studies have indicated that NK cells have achieved good efficacy in treating and killing tumours, especially in colon cancer, and have good prospects for transformation (42, 43). Ma (44) constructed an immune-related module that consists of SYP and 13 other genes, which may be innovative biomarkers for the prediction of colon cancer prognosis and response to immunological therapy. Opinion is divided over the importance of WNT5A—an essential protein of the non-canonical Wnt/β-catenin signalling pathway—in colon cancer (45). Cecilia (46), Cheng (47), and Li (48) argued that WNT5A is a protective factor that delays disease progression in colon cancer patients. In contrast, Elvira (49) asserts that high WNT5A expression could induce colon cancer cell migration and invasion. POLA1 has been reported to influence the occurrence and development of tumours (50–52), and its expression can be suppressed by the novel compound ST1926 (53) and antitumor toxin CD437 (54). NCAPG has been reported as an oncogene in liver cancer (55), gastric cancer (56), breast cancer (57), and other tumours but has not yet been studied in CRC. **Figure 10** combined with **Figure 11** shows that the correlations between the ENOX1, ZC3H10, and SAMD4A genes and the M2 macrophages and TAMs were much better than those with the M1 macrophages. Existing studies generally believe that macrophage polarisation and TAMs are extremely important for tumour immune evasion and the establishment of an immune microenvironment. Hence, it could conceivably be hypothesised that these genes help colorectal cancer cells escape immune monitoring and clearance to a certain extent. At present, there is very little research on these three genes related to immunity; this is a new research direction worth exploring. The NADH oxidase ENOX1 targets tumour vasculature and can be used in tumour treatment (58, 59). Coincidentally, Zhou et al. (60) also reported SAMD4A as a novel breast tumour angiogenesis suppressor in breast cancer. ZC3H10 regulates lipid metabolism and may also offer novel routes toward treatments for obesity (61).

## Conclusion

In this study, we discovered novel circRNAs derived from the RPPH1 gene in CRC. The circRNAs work with other differentially expressed RNAs to constitute a pair of immunoregulatory circRNA–miRNA–mRNA and circRNA–protein networks that act upon CRC. We further explored the relationship between regulated genes and immune cell behaviours. Futher research is required to elucidate the functional behaviour of these two molecular networks. We believe that the interaction networks of these eight circRNAs, five target genes, and four immune-related proteins offer a new paradigm for the understanding of CRC pathogenesis and could lead to entirely new approaches to treatment.

## Data Availability Statement

The original contributions presented in the study are included in the article/**Supplementary Material**, further inquiries can be directed to the corresponding author/s.

## Author Contributions

ZF, LLi, and XS drafted the manuscript. WC, QZ, and YZ participated in the data processing. LLu, AW, and YT participated in qRT-PCR assays. ZL and YC designed the study and revised the manuscript. All authors contributed to the article and approved the submitted version.

## Funding

This work was supported by the National Natural Science Foundation of China (No. 81860428), the Leading Talents Program of Jiangxi (20213BCJL22050), the Youth Science Foundation of Jiangxi Province (No. 20202BABL216051), the Science and Technology Plan of Health Commission of Jiangxi Province (No. 20191026), the Spark Promotion Plan of Grassroots Health Appropriate Technology of the Health Commission of Jiangxi Province (No. 68120198012), the Project of Science and Technology Department of Jiangxi Province (No. 20203BBGL73187), and the Traditional Chinese Medicine Research Project of Jiangxi Province (No. 2019A185).

## Conflict of Interest

The authors declare that the research was conducted in the absence of any commercial or financial relationships that could be construed as a potential conflict of interest.

## Publisher’s Note

All claims expressed in this article are solely those of the authors and do not necessarily represent those of their affiliated organizations, or those of the publisher, the editors and the reviewers. Any product that may be evaluated in this article, or claim that may be made by its manufacturer, is not guaranteed or endorsed by the publisher.

## Supplementary Material

Supplementary Figure S1 Correlation between ENOX1 expression and markers of immune cells as analysed through GEPIA2. **(A)** for B cell, **(B)** for CD4+ T cell, **(C)** for CD8+ T cell, **(D)** for dendritic cell, **(E)** for effector regulatory T (Treg) cell, **(F)** for eosinophils, **(G)** for exhausted T cell, **(H)** for mast cell, **(I)** for monocyte, **(J)** for neutrophils, **(K)** for natural killer (NK) cell, **(L)** for resting regulatory T (Treg) cell, **(M)** for T follicular helper (Tfh) cell, **(N)** for T helper type 1 (Th1) cell, **(O)** for Th2 cell, **(P)** for Th17 cell.

Supplementary Figure S2 Correlation between NCAM1 expression and markers of immune cells as analysed through GEPIA2. **(A)** for B cell, **(B)** for CD4+ T cell, **(C)** for CD8+ T cell, **(D)** for dendritic cell, **(E)** for effector regulatory T (Treg) cell, **(F)** for eosinophils, **(G)** for exhausted T cell, **(H)** for mast cell, **(I)** for monocyte, **(J)** for neutrophils, **(K)** for natural killer (NK) cell, **(L)** for resting regulatory T (Treg) cell, **(M)** for T follicular helper (Tfh) cell, **(N)** for T helper type 1 (Th1) cell, **(O)** for Th2 cell, **(P)** for Th17 cell.

Supplementary Figure S3 Correlation between SAMD4A expression and markers of immune cells as analysed through GEPIA2. **(A)** for B cell, **(B)** for CD4+ T cell, **(C)** for CD8+ T cell, **(D)** for dendritic cell, **(E)** for effector regulatory T (Treg) cell, **(F)** for eosinophils, **(G)** for exhausted T cell, **(H)** for mast cell, **(I)** for monocyte, **(J)** for neutrophils, **(K)** for natural killer (NK) cell, **(L)** for resting regulatory T (Treg) cell, **(M)** for T follicular helper (Tfh) cell, **(N)** for T helper type 1 (Th1) cell, **(O)** for Th2 cell, **(P)** for Th17 cell.

Supplementary Figure S4 Correlation between ZC3H10 expression and markers of immune cells as analysed through GEPIA2. **(A)** for B cell, **(B)** for CD4+ T cell, **(C)** for CD8+ T cell, **(D)** for dendritic cell, **(E)** for effector regulatory T (Treg) cell, **(F)** for eosinophils, **(G)** for exhausted T cell, **(H)** for mast cell, **(I)** for monocyte, **(J)** for neutrophils, **(K)** for natural killer (NK) cell, **(L)** for resting regulatory T (Treg) cell, **(M)** for T follicular helper (Tfh) cell, **(N)** for T helper type 1 (Th1) cell, **(O)** for Th2 cell, and **(P)** for Th17 cell.

Supplementary Figure S5 Melting curve of eight circular RNAs (circRNAs) and GAPDH in colorectal cancer (CRC). **(A)** for hsa_circ_0000511, **(B)** for hsa_circ_0000512, **(C)** for hsa_circ_0000514, **(D)** for hsa_circ_0000515, **(E)** for hsa_circ_0000517, **(F)** for hsa_circ_0000518, **(G)** for hsa_circ_0000519, **(H)** for hsa_circ_0000520, and **(I)** for GAPDH.

Supplementary Figure S6 Relative miRNAs and mRNA expression in colorectal cancer (CRC) cells and NCM-460 normal cell. **(A)** for miRNA-296-5p, **(B)** for miRNA-1976, **(C)** for miRNA-1306-5p, **(D)** for miRNA-326, **(E)** for miRNA-1296-5p, **(F)** for NCAM1, **(G)** for CD44, **(H)** for SYP, **(I)** for NCAPG, **(J)** for WNT5A, and **(K)** for POLA1.

Supplementary Table S1 Primers for miRNAs and mRNAs amplification in the competing endogenous RNA (ceRNA) network.

Supplementary Table S2 The protein binding with eight circular RNAs (circRNAs) from the RBPmap tool.

Supplementary Table S3 Expression levels of eight circular RNAs (circRNAs) in colorectal cancer (CRC) cells as detected by qRT-PCR assays.

## References

[1] Sung H Ferlay J Siegel RL Laversanne M Soerjomataram I Jemal A. Global Cancer Statistics 2020: GLOBOCAN Estimates of Incidence and Mortality Worldwide for 36 Cancers in 185 Countries. CA Cancer J Clin (2021) 71(3):209–49. doi: 10.3322/caac.21660 33538338

[2] Gao Q Li XX Xu YM Zhang JZ Rong SD Qin YQ. Ire1α-Targeting Downregulates ABC Transporters and Overcomes Drug Resistance of Colon Cancer Cells. Cancer Lett (2020) 476:67–74. doi: 10.1016/j.canlet.2020.02.007 32061752

[3] Hansen TB Jensen TI Clausen BH Bramsen JB Finsen B Damgaard CK. Natural RNA Circles Function as Efficient microRNA Sponges. Nature (2013) 495(7441):384–8. doi: 10.1038/nature11993 23446346

[4] Okholm TLH Sathe S Park SS Kamstrup AB Rasmussen AM Shankar A. Transcriptome-Wide Profiles of Circular RNA and RNA-Binding Protein Interactions Reveal Effects on Circular RNA Biogenesis and Cancer Pathway Expression. Genome Med (2020) 12(1):112. doi: 10.1186/s13073-020-00812-8 33287884 PMC7722315

[5] Lei M Zheng G Ning Q Zheng J Dong D. Translation and Functional Roles of Circular RNAs in Human Cancer. Mol Cancer (2020) 19(1):30. doi: 10.1186/s12943-020-1135-7 32059672 PMC7023758

[6] Peng L Sang H Wei S Li Y Jin D Zhu X. Circcul2 Regulates Gastric Cancer Malignant Transformation and Cisplatin Resistance by Modulating Autophagy Activation *via* miR-142-3p/ROCK2. Mol Cancer (2020) 19(1):156. doi: 10.1186/s12943-020-01270-x 33153478 PMC7643398

[7] Yang H Li X Meng Q Sun H Wu S Hu W. CircPTK2 (Hsa_Circ_0005273) as a Novel Therapeutic Target for Metastatic Colorectal Cancer. Mol Cancer (2020) 19(1):13. doi: 10.1186/s12943-020-1139-3 31973707 PMC6977296

[8] Zhou J Tang Z Gao S Li C Feng Y Zhou X. Tumor-Associated Macrophages: Recent Insights and Therapies. Front Oncol (2020) 10:188. doi: 10.3389/fonc.2020.00188 32161718 PMC7052362

[9] Lan J Sun L Xu F Liu L Hu F Song D. M2 Macrophage-Derived Exosomes Promote Cell Migration and Invasion in Colon Cancer. Cancer Res (2019) 79(1):146–58. doi: 10.1158/0008-5472.Can-18-0014 30401711

[10] Cheng Y Zhu Y Xu J Yang M Chen P Xu W. PKN2 in Colon Cancer Cells Inhibits M2 Phenotype Polarization of Tumor-Associated Macrophages *via* Regulating DUSP6-Erk1/2 Pathway. Mol Cancer (2018) 17(1):13. doi: 10.1186/s12943-017-0747-z 29368606 PMC5784528

[11] Wu Y Cheng K Liang W Wang X. lncRNA RPPH1 Promotes Non-Small Cell Lung Cancer Progression Through the miR-326/WNT2B Axis. Oncol Lett (2020) 20(4):105. doi: 10.3892/ol.2020.11966 32831924 PMC7439152

[12] Yue K Ma JL Jiang T Yue J Sun SK Shen JL. LncRNA RPPH1 Predicts Poor Prognosis and Regulates Cell Proliferation and Migration by Repressing P21 Expression in Gastric Cancer. Eur Rev Med Pharmacol Sci (2020) 24(21):11072–80. doi: 10.26355/eurrev_202011_23593 33215423

[13] Liang ZX Liu HS Wang FW Xiong L Zhou C Hu T. LncRNA RPPH1 Promotes Colorectal Cancer Metastasis by Interacting With TUBB3 and by Promoting Exosomes-Mediated Macrophage M2 Polarization. Cell Death Dis (2019) 10(11):829. doi: 10.1038/s41419-019-2077-0 31685807 PMC6828701

[14] Todaro M Gaggianesi M Catalano V Benfante A Iovino F Biffoni M. CD44v6 Is a Marker of Constitutive and Reprogrammed Cancer Stem Cells Driving Colon Cancer Metastasis. Cell Stem Cell (2014) 14(3):342–56. doi: 10.1016/j.stem.2014.01.009 24607406

[15] Ma L Dong L Chang P. CD44v6 Engages in Colorectal Cancer Progression. Cell Death Dis (2019) 10(1):30. doi: 10.1038/s41419-018-1265-7 30631039 PMC6328617

[16] Melsen JE Lugthart G Lankester AC Schilham MW. Human Circulating and Tissue-Resident CD56(bright) Natural Killer Cell Populations. Front Immunol (2016) 7:262. doi: 10.3389/fimmu.2016.00262 27446091 PMC4927633

[17] Gharagozloo M Rezaei A Kalantari H Bahador A Hassannejad N Maracy M. Decline in Peripheral Blood NKG2D+CD3+CD56+ NKT Cells in Metastatic Colorectal Cancer Patients. Bratisl Lek Listy (2018) 119(1):6–11. doi: 10.4149/bll_2018_002 29405723

[18] Chen Z Ren R Wan D Wang Y Xue X Jiang M. Hsa_circ_101555 Functions as a Competing Endogenous RNA of miR-597-5p to Promote Colorectal Cancer Progression. Oncogene (2019) 38(32):6017–34. doi: 10.1038/s41388-019-0857-8 31300733

[19] Ritchie ME Phipson B Wu D Hu Y Law CW Shi W. Limma Powers Differential Expression Analyses for RNA-Sequencing and Microarray Studies. Nucleic Acids Res (2015) 43(7):e47. doi: 10.1093/nar/gkv007 25605792 PMC4402510

[20] Li JH Liu S Zhou H Qu LH Yang JH. Starbase V2.0: Decoding miRNA-ceRNA, miRNA-ncRNA and Protein-RNA Interaction Networks From Large-Scale CLIP-Seq Data. Nucleic Acids Res (2014) 42(Database issue):D92–7. doi: 10.1093/nar/gkt1248 PMC3964941 24297251

[21] Liu M Wang Q Shen J Yang BB Ding X. Circbank: A Comprehensive Database for circRNA With Standard Nomenclature. RNA Biol (2019) 16(7):899–905. doi: 10.1080/15476286.2019.1600395 31023147 PMC6546381

[22] Sticht C de la Torre C Parveen A Gretz N. Mirwalk: An Online Resource for Prediction of microRNA Binding Sites. PloS One (2018) 13(10):e0206239. doi: 10.1371/journal.pone.0206239 30335862 PMC6193719

[23] Shannon P Markiel A Ozier O Baliga NS Wang JT Ramage D. Cytoscape: A Software Environment for Integrated Models of Biomolecular Interaction Networks. Genome Res (2003) 13(11):2498–504. doi: 10.1101/gr.1239303 PMC403769 14597658

[24] Szklarczyk D Gable AL Lyon D Junge A Wyder S Huerta-Cepas J. STRING V11: Protein-Protein Association Networks With Increased Coverage, Supporting Functional Discovery in Genome-Wide Experimental Datasets. Nucleic Acids Res (2019) 47(D1):D607–13. doi: 10.1093/nar/gky1131 30476243 PMC6323986

[25] Liu CJ Hu FF Xia MX Han L Zhang Q. Guo AY GSCALite: A Web Server for Gene Set Cancer Analysis. Bioinformatics (2018) 34(21):3771–2. doi: 10.1093/bioinformatics/bty411 29790900

[26] Paz I Kosti I Ares M Jr Cline M. Mandel-Gutfreund Y RBPmap: A Web Server for Mapping Binding Sites of RNA-Binding Proteins. Nucleic Acids Res (2014) 42(Web Server issue):W361–367. doi: 10.1093/nar/gku406 PMC4086114 24829458

[27] Li T Fu J Zeng Z Cohen D Li J Chen Q. TIMER2.0 for Analysis of Tumor-Infiltrating Immune Cells. Nucleic Acids Res (2020) 48(W1):W509–14. doi: 10.1093/nar/gkaa407 32442275 PMC7319575

[28] Chen J Yang X Liu R Wen C Wang H Huang L. Circular RNA GLIS2 Promotes Colorectal Cancer Cell Motility *via* Activation of the NF-κb Pathway. Cell Death Dis (2020) 11(9):788. doi: 10.1038/s41419-020-02989-7 32968054 PMC7511409

[29] Xie J Chen Q Zhou P Fan W. Circular RNA Hsa_Circ_0000511 Improves Epithelial Mesenchymal Transition of Cervical Cancer by Regulating Hsa-Mir-296-5p/HMGA1. J Immunol Res (2021) 2021:9964538. doi: 10.1155/2021/9964538 34136582 PMC8175136

[30] Cai F Fu W Tang L Tang J Sun J Fu G. Hsa_circ_0000515 Is a Novel Circular RNA Implicated in the Development of Breast Cancer Through Its Regulation of the microRNA-296-5p/CXCL10 Axis. FEBS J (2021) 288(3):861–83. doi: 10.1111/febs.15373 32446265

[31] He S Guo Z Kang Q Wang X Han X. Circular RNA Hsa_Circ_0000517 Modulates Hepatocellular Carcinoma Advancement *via* the miR-326/SMAD6 Axis. Cancer Cell Int (2020) 20:360. doi: 10.1186/s12935-020-01447-w 32774154 PMC7397604

[32] He S Yang J Jiang S Li Y Han X. Circular RNA Circ_0000517 Regulates Hepatocellular Carcinoma Development *via* miR-326/IGF1R Axis. Cancer Cell Int (2020) 20:404. doi: 10.1186/s12935-020-01496-1 32863763 PMC7448484

[33] Wang X Wang X Li W Zhang Q Chen J Chen T. Up-Regulation of Hsa_Circ_0000517 Predicts Adverse Prognosis of Hepatocellular Carcinoma. Front Oncol (2019) 9:1105. doi: 10.3389/fonc.2019.01105 31750237 PMC6842961

[34] Jiang J Lin H Shi S Hong Y Bai X Cao X. Hsa_circRNA_0000518 Facilitates Breast Cancer Development *via* Regulation of the miR-326/FGFR1 Axis. Thorac Cancer (2020) 11(11):3181–92. doi: 10.1111/1759-7714.13641 PMC7606003 33000910

[35] Sun H Tang W Rong D Jin H Fu K Zhang W. Hsa_circ_0000520, a Potential New Circular RNA Biomarker, Is Involved in Gastric Carcinoma. Cancer biomark (2018) 21(2):299–306. doi: 10.3233/cbm-170379 29103021 PMC13078270

[36] Lv X Li P Wang J Gao H Hei Y Zhang J. Hsa_Circ_0000520 Influences Herceptin Resistance in Gastric Cancer Cells Through PI3K-Akt Signaling Pathway. J Clin Lab Anal (2020) 34(10):e23449. doi: 10.1002/jcla.23449 32701211 PMC7595902

[37] Zang H Li Y Zhang X Huang G. Blocking Circ_0000520 Suppressed Breast Cancer Cell Growth, Migration and Invasion Partially *via* miR-1296/SP1 Axis Both *In Vitro* and *In Vivo* . Cancer Manag Res (2020) 12:7783–95. doi: 10.2147/cmar.S251666 PMC7457856 32922078

[38] Zhou Y Ma G Peng S Tuo M Li Y Qin X. Circ_0000520 Contributes to Triple-Negative Breast Cancer Progression Through Mediating the miR-1296/ZFX Axis. Thorac Cancer (2021) 12(18):2427–38. doi: 10.1111/1759-7714.14085 PMC8447912 34324278

[39] Zheng Q Zhang J Zhang T Liu Y Du X Dai X. Hsa_circ_0000520 Overexpression Increases CDK2 Expression *via* miR-1296 to Facilitate Cervical Cancer Cell Proliferation. J Transl Med (2021) 19(1):314. doi: 10.1186/s12967-021-02953-9 34284793 PMC8290540

[40] Zhang J Cai R Zhang Y Wang X. Involvement of a Novel circularRNA, Hsa_Circ_0000520, Attenuates Tumorigenesis of Cervical Cancer Cell Through Competitively Binding With miR-146b-3p. J Cell Mol Med (2020) 24(15):8480–90. doi: 10.1111/jcmm.15414 PMC7412397 32592222

[41] Pothuraju R Rachagani S Krishn SR Chaudhary S Nimmakayala RK Siddiqui JA. Molecular Implications of MUC5AC-CD44 Axis in Colorectal Cancer Progression and Chemoresistance. Mol Cancer (2020) 19(1):37. doi: 10.1186/s12943-020-01156-y 32098629 PMC7041280

[42] Huang YW Lin CW Pan P Shan T Echeveste CE Mo YY. Black Raspberries Suppress Colorectal Cancer by Enhancing Smad4 Expression in Colonic Epithelium and Natural Killer Cells. Front Immunol (2020) 11:570683. doi: 10.3389/fimmu.2020.570683 33424832 PMC7793748

[43] Zhang Q Bi J Zheng X Chen Y Wang H Wu W. Blockade of the Checkpoint Receptor TIGIT Prevents NK Cell Exhaustion and Elicits Potent Anti-Tumor Immunity. Nat Immunol (2018) 19(7):723–32. doi: 10.1038/s41590-018-0132-0 29915296

[44] Ma XB Xu YY Zhu MX Wang L. Prognostic Signatures Based on Thirteen Immune-Related Genes in Colorectal Cancer. Front Oncol (2020) 10:591739. doi: 10.3389/fonc.2020.591739 33680920 PMC7935549

[45] Kikuchi A Yamamoto H Sato A Matsumoto S. Wnt5a: Its Signalling, Functions and Implication in Diseases. Acta Physiol (Oxf) (2012) 204(1):17–33. doi: 10.1111/j.1748-1716.2011.02294.x 21518267

[46] Lund CM Dyhl-Polk A Nielsen DL Riis LB. Wnt5a Expression and Prognosis in Stage II-III Colon Cancer. Transl Oncol (2021) 14(1):100892. doi: 10.1016/j.tranon.2020.100892 33045677 PMC7553443

[47] Cheng R Sun B Liu Z Zhao X Qi L Li Y. Wnt5a Suppresses Colon Cancer by Inhibiting Cell Proliferation and Epithelial-Mesenchymal Transition. J Cell Physiol (2014) 229(12):1908–17. doi: 10.1002/jcp.24566 24464650

[48] Li Q Chen H. Silencing of Wnt5a During Colon Cancer Metastasis Involves Histone Modifications. Epigenetics (2012) 7(6):551–8. doi: 10.4161/epi.20050 PMC3398984 22522911

[49] Bakker ER Das AM Helvensteijn W Franken PF Swagemakers S van der Valk MA. Wnt5a Promotes Human Colon Cancer Cell Migration and Invasion But Does Not Augment Intestinal Tumorigenesis in Apc1638N Mice. Carcinogenesis (2013) 34(11):2629–38. doi: 10.1093/carcin/bgt215 23764752

[50] Rogers RF Walton MI Cherry DL Collins I Clarke PA Garrett MD. CHK1 Inhibition Is Synthetically Lethal With Loss of B-Family DNA Polymerase Function in Human Lung and Colorectal Cancer Cells. Cancer Res (2020) 80(8):1735–47. doi: 10.1158/0008-5472.Can-19-1372 PMC7611445 32161100

[51] Liu J Liu S Yang X. Construction of Gene Modules and Analysis of Prognostic Biomarkers for Cervical Cancer by Weighted Gene Co-Expression Network Analysis. Front Oncol (2021) 11:542063. doi: 10.3389/fonc.2021.542063 33816217 PMC8016394

[52] Cui L Xue H Wen Z Lu Z Liu Y Zhang Y. Prognostic Roles of Metabolic Reprogramming-Associated Genes in Patients With Hepatocellular Carcinoma. Aging (Albany NY) (2020) 12(21):22199–219. doi: 10.18632/aging.104122 PMC7695384 33188160

[53] Abdel-Samad R Aouad P Gali-Muhtasib H Sweidan Z Hmadi R Kadara H. Mechanism of Action of the Atypical Retinoid ST1926 in Colorectal Cancer: DNA Damage and DNA Polymerase α. Am J Cancer Res (2018) 8(1):39–55. 29416919 PMC5794720

[54] Han T Goralski M Capota E Padrick SB Kim J Xie Y. The Antitumor Toxin CD437 Is a Direct Inhibitor of DNA Polymerase α. Nat Chem Biol (2016) 12(7):511–5. doi: 10.1038/nchembio.2082 PMC4912453 27182663

[55] Gong C Ai J Fan Y Gao J Liu W Feng Q. NCAPG Promotes The Proliferation Of Hepatocellular Carcinoma Through PI3K/AKT Signaling. Onco Targets Ther (2019) 12:8537–52. doi: 10.2147/ott.S217916 PMC6801502 31802891

[56] Sun DP Lin CC Hung ST Kuang YY Hseu YC Fang CL. Aberrant Expression of NCAPG Is Associated With Prognosis and Progression of Gastric Cancer. Cancer Manag Res (2020) 12:7837–46. doi: 10.2147/cmar.S248318 PMC7457733 32922082

[57] Jiang L Ren L Chen H Pan J Zhang Z Kuang X. NCAPG Confers Trastuzumab Resistance *via* Activating SRC/STAT3 Signaling Pathway in HER2-Positive Breast Cancer. Cell Death Dis (2020) 11(7):547. doi: 10.1038/s41419-020-02753-x 32683421 PMC7368860

[58] Geng L Rachakonda G Morré DJ Morré DM Crooks PA Sonar VN. Indolyl-Quinuclidinols Inhibit ENOX Activity and Endothelial Cell Morphogenesis While Enhancing Radiation-Mediated Control of Tumor Vasculature. FASEB J (2009) 23(9):2986–95. doi: 10.1096/fj.09-130005 PMC2796904 19395476

[59] Venkateswaran A Friedman DB Walsh AJ Skala MC Sasi S Rachakonda G. The Novel Antiangiogenic VJ115 Inhibits the NADH Oxidase ENOX1 and Cytoskeleton-Remodeling Proteins. Invest New Drugs (2013) 31(3):535–44. doi: 10.1007/s10637-012-9884-9 PMC3553230 23054211

[60] Zhou M Wang B Li H Han J Li A Lu W. RNA-Binding Protein SAMD4A Inhibits Breast Tumor Angiogenesis by Modulating the Balance of Angiogenesis Program. Cancer Sci (2021) 112(9):3835–45. doi: 10.1111/cas.15053 PMC8409301 34219323

[61] Yi D Nguyen HP Dinh J Viscarra JA Xie Y Lin F. Dot1l Interacts With Zc3h10 to Activate Ucp1 and Other Thermogenic Genes. Elife (2020) 27(9):e59990. doi: 10.7554/eLife.59990 PMC7661038 33107819

