# Supplementary figures and images for: Identification of Circular RNA-Based Immunomodulatory Networks in Colorectal Cancer
Source: Front Oncol. 2022 Jan 27;11:779706. doi: 10.3389/fonc.2021.779706 (PMC8833313; doi:10.3389/fonc.2021.779706)

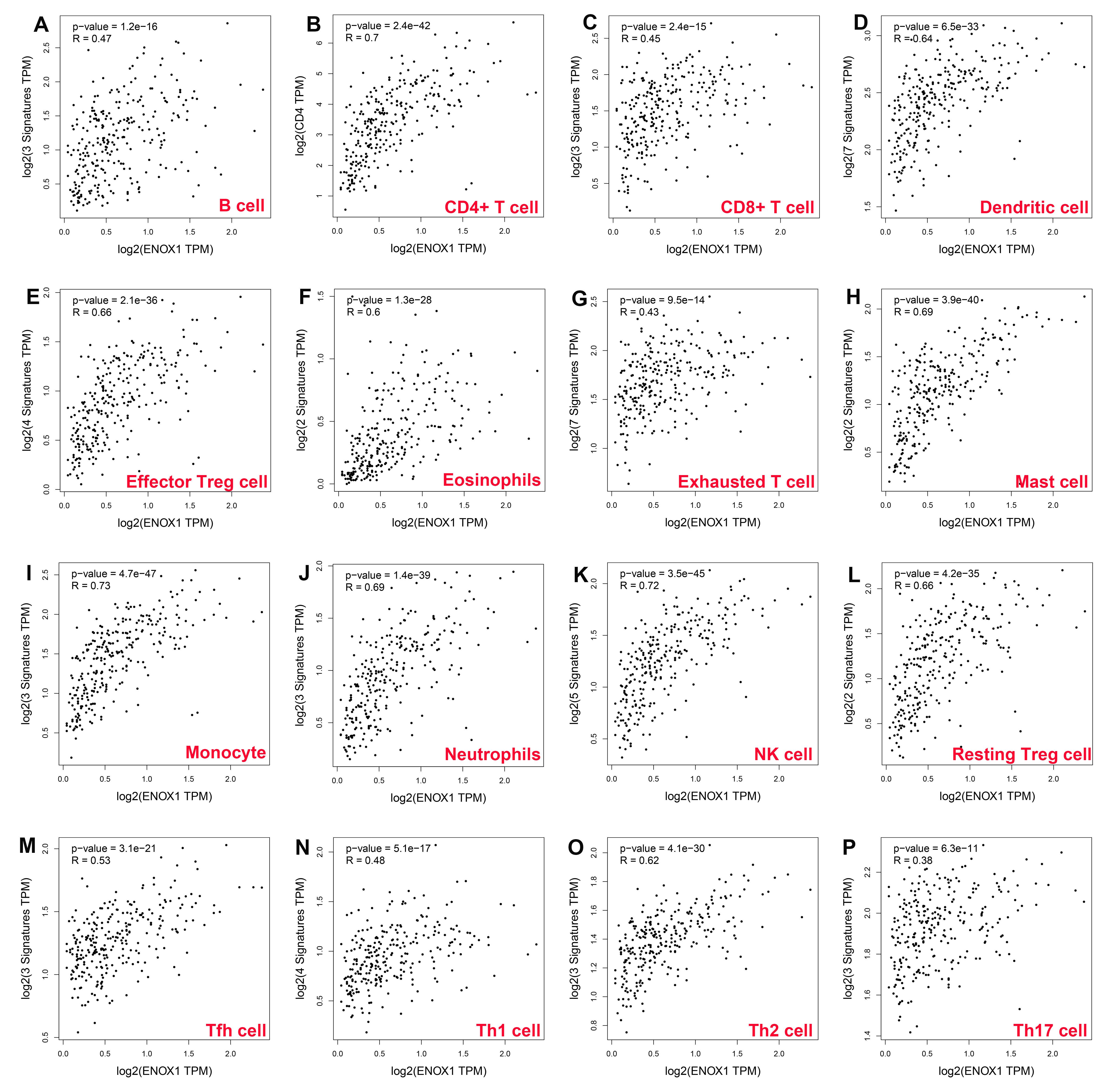

Supplement: Supplementary Figure S1 — Correlation between ENOX1 expression and markers of immune cells as analysed through GEPIA2. (A) for B cell, (B) for CD4+ T cell, (C) for CD8+ T cell, (D) for dendritic cell, (E) for effector regulatory T (Treg) cell, (F) for eosinophils, (G) for exhausted T cell, (H) for mast cell, (I) for monocyte, (J) for neutrophils, (K) for natural killer (NK) cell, (L) for resting regulatory T (Treg) cell, (M) for T follicular helper (Tfh) cell, (N) for T helper type 1 (Th1) cell, (O) for Th2 cell, (P) for Th17 cell. [file Image_1.tif]

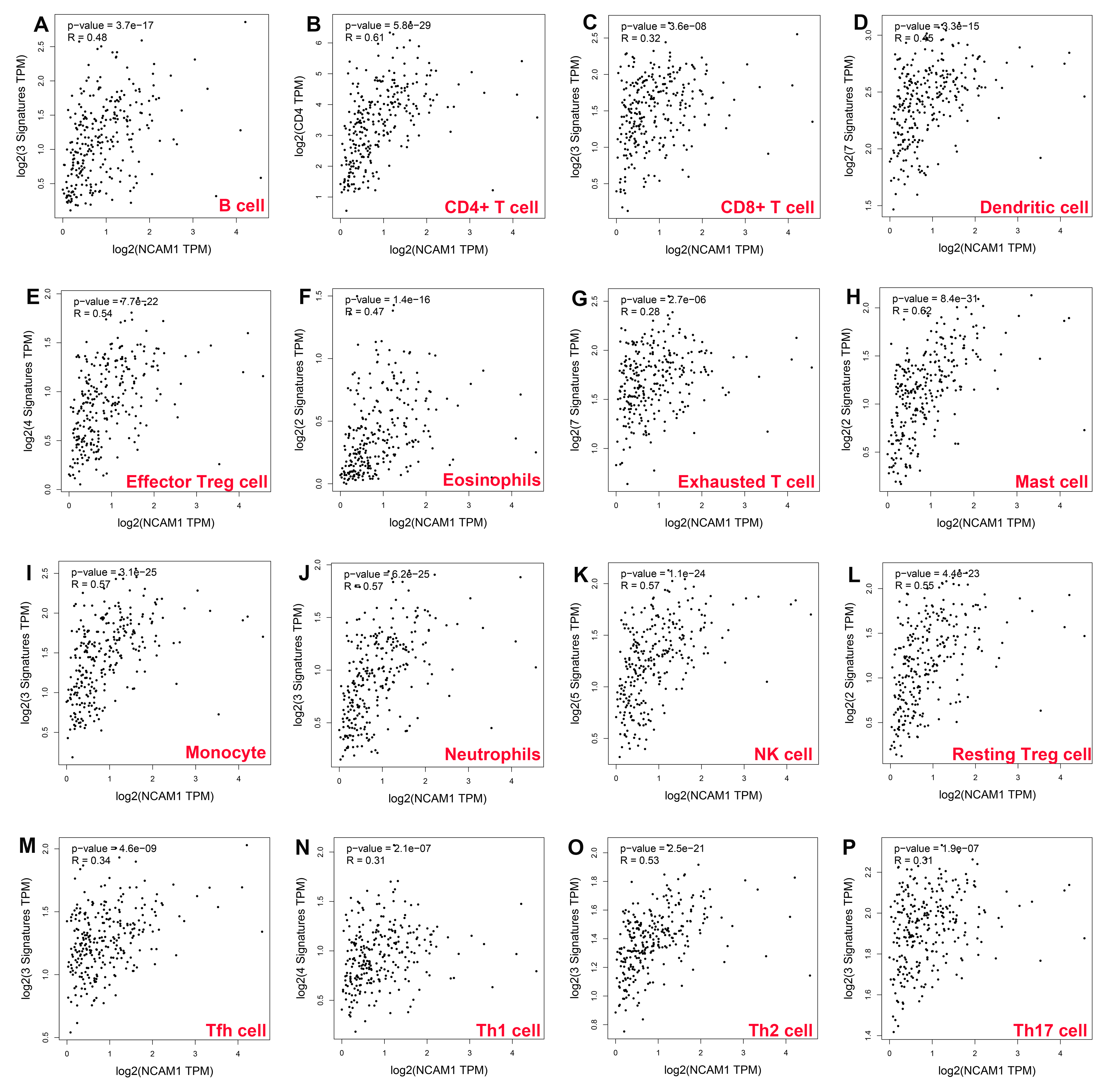

Supplement: Supplementary Figure S2 — Correlation between NCAM1 expression and markers of immune cells as analysed through GEPIA2. (A) for B cell, (B) for CD4+ T cell, (C) for CD8+ T cell, (D) for dendritic cell, (E) for effector regulatory T (Treg) cell, (F) for eosinophils, (G) for exhausted T cell, (H) for mast cell, (I) for monocyte, (J) for neutrophils, (K) for natural killer (NK) cell, (L) for resting regulatory T (Treg) cell, (M) for T follicular helper (Tfh) cell, (N) for T helper type 1 (Th1) cell, (O) for Th2 cell, (P) for Th17 cell. [file Image_2.tif]

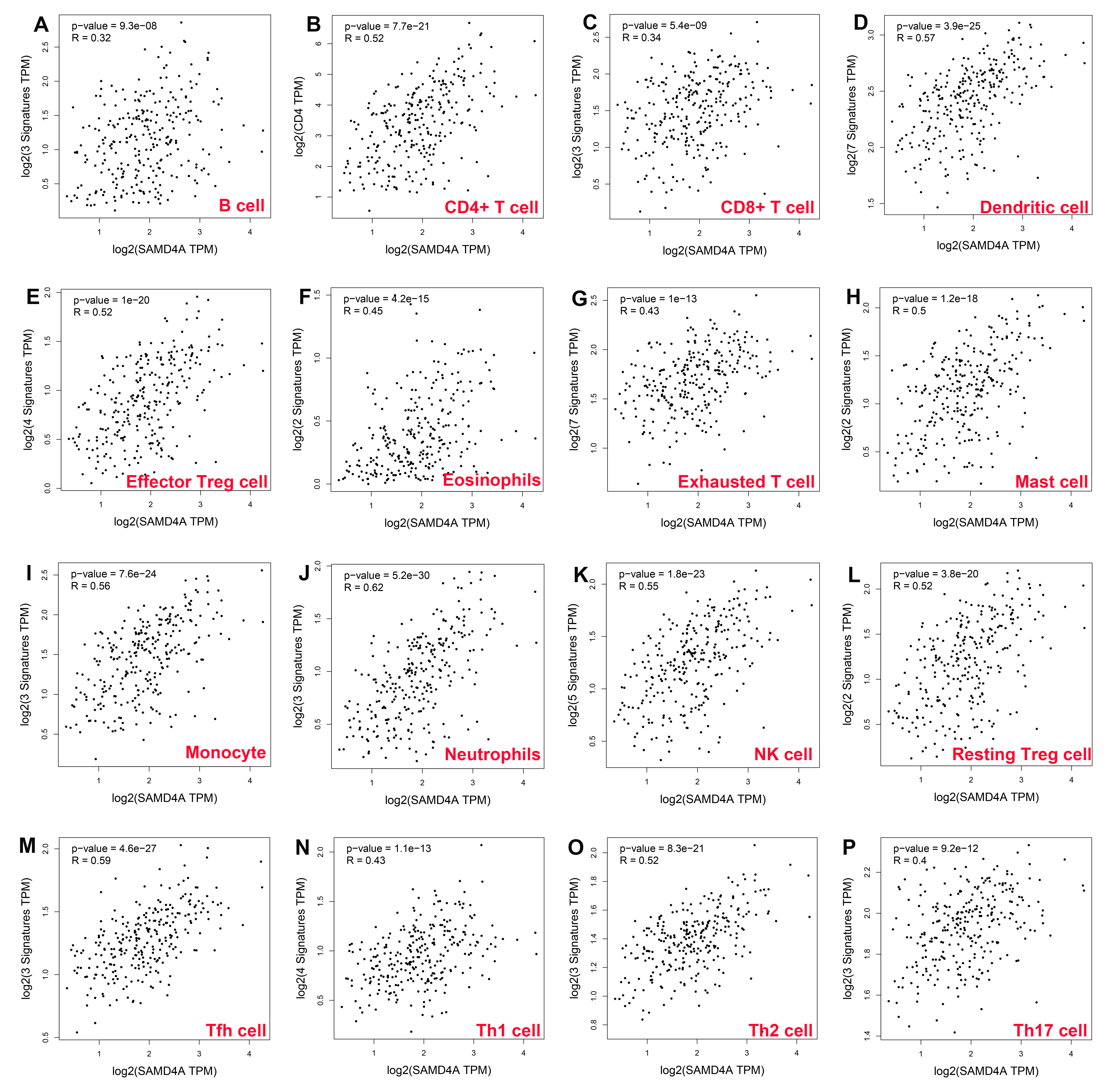

Supplement: Supplementary Figure S3 — Correlation between SAMD4A expression and markers of immune cells as analysed through GEPIA2. (A) for B cell, (B) for CD4+ T cell, (C) for CD8+ T cell, (D) for dendritic cell, (E) for effector regulatory T (Treg) cell, (F) for eosinophils, (G) for exhausted T cell, (H) for mast cell, (I) for monocyte, (J) for neutrophils, (K) for natural killer (NK) cell, (L) for resting regulatory T (Treg) cell, (M) for T follicular helper (Tfh) cell, (N) for T helper type 1 (Th1) cell, (O) for Th2 cell, (P) for Th17 cell. [file Image_3.tif]

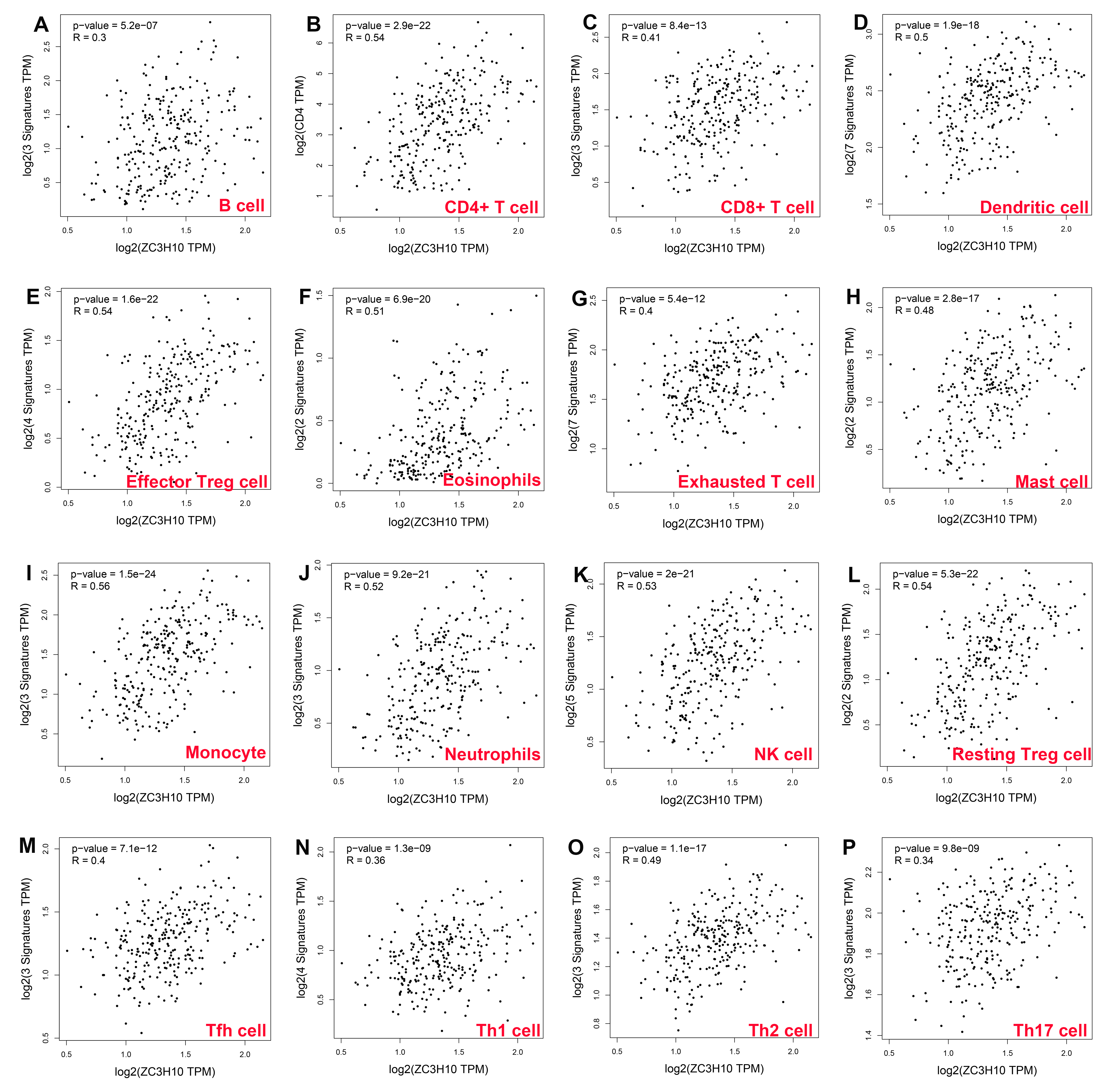

Supplement: Supplementary Figure S4 — Correlation between ZC3H10 expression and markers of immune cells as analysed through GEPIA2. (A) for B cell, (B) for CD4+ T cell, (C) for CD8+ T cell, (D) for dendritic cell, (E) for effector regulatory T (Treg) cell, (F) for eosinophils, (G) for exhausted T cell, (H) for mast cell, (I) for monocyte, (J) for neutrophils, (K) for natural killer (NK) cell, (L) for resting regulatory T (Treg) cell, (M) for T follicular helper (Tfh) cell, (N) for T helper type 1 (Th1) cell, (O) for Th2 cell, and (P) for Th17 cell. [file Image_4.tif]

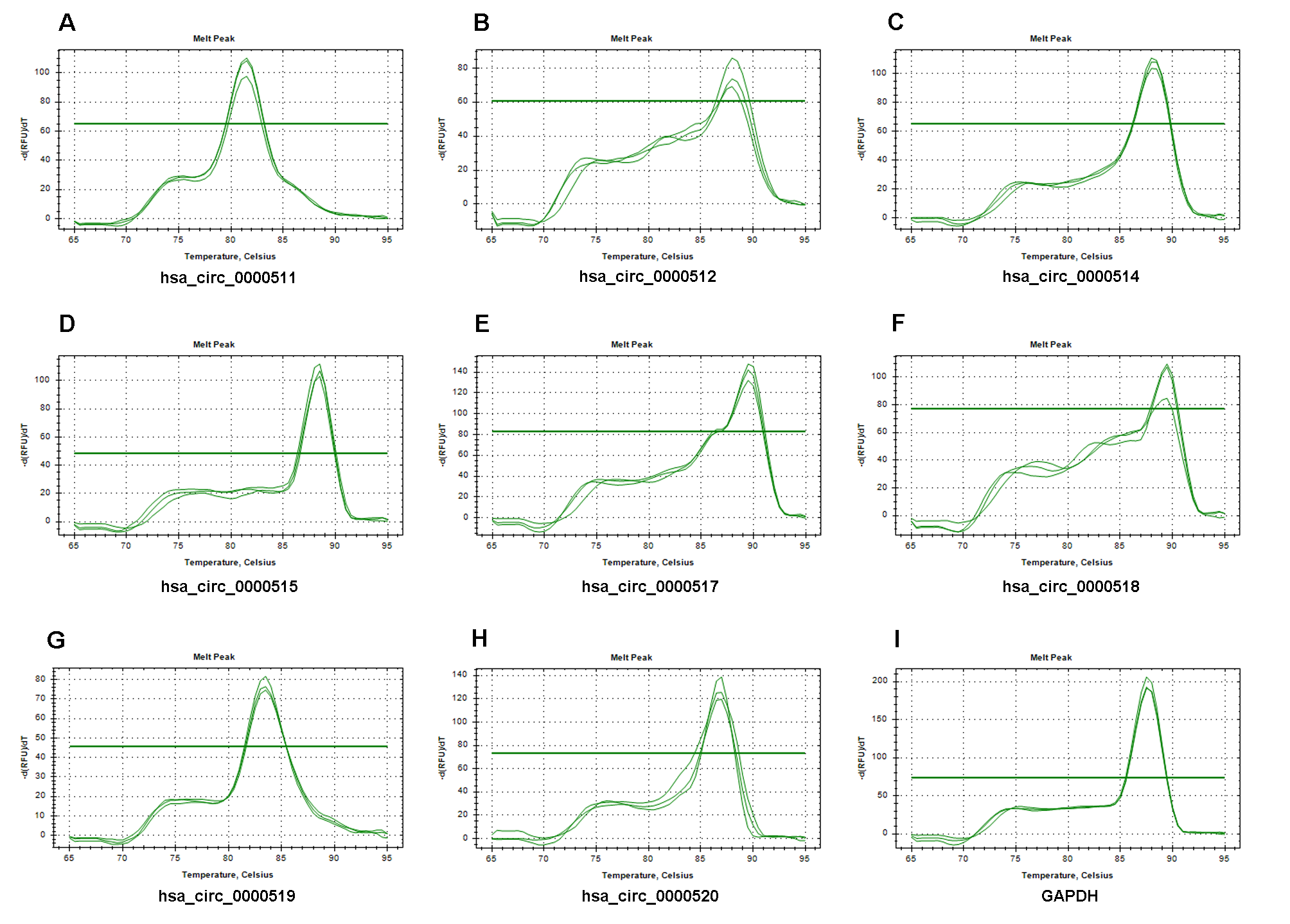

Supplement: Supplementary Figure S5 — Melting curve of eight circular RNAs (circRNAs) and GAPDH in colorectal cancer (CRC). (A) for hsa_circ_0000511, (B) for hsa_circ_0000512, (C) for hsa_circ_0000514, (D) for hsa_circ_0000515, (E) for hsa_circ_0000517, (F) for hsa_circ_0000518, (G) for hsa_circ_0000519, (H) for hsa_circ_0000520, and (I) for GAPDH. [file Image_5.tif]

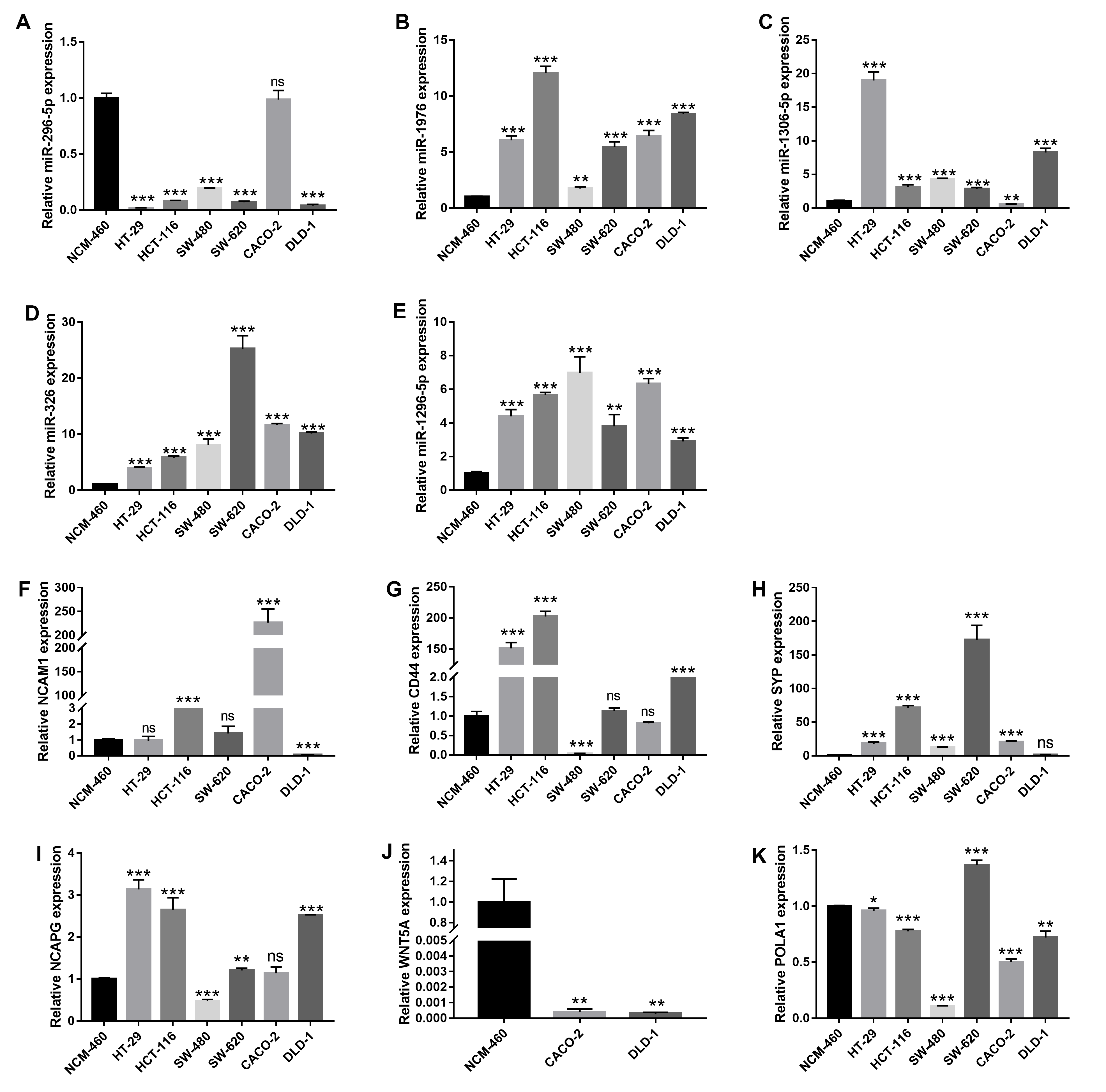

Supplement: Supplementary Figure S6 — Relative miRNAs and mRNA expression in colorectal cancer (CRC) cells and NCM-460 normal cell. (A) for miRNA-296-5p, (B) for miRNA-1976, (C) for miRNA-1306-5p, (D) for miRNA-326, (E) for miRNA-1296-5p, (F) for NCAM1, (G) for CD44, (H) for SYP, (I) for NCAPG, (J) for WNT5A, and (K) for POLA1. [file Image_6.tif]
