# Supplementary material for: Identification of Circular RNA-Based Immunomodulatory Networks in Colorectal Cancer
Source: Front Oncol. 2022 Jan 27;11:779706. doi: 10.3389/fonc.2021.779706 (PMC8833313; doi:10.3389/fonc.2021.779706)
Supplement: Supplementary Table S1 — Primers for miRNAs and mRNAs amplification in the competing endogenous RNA (ceRNA) network. [file Table_1.docx]

**Table S1: miRNAs and mRNAs primers**

| Primer name | sequence (5'to 3') |
| --- | --- |
| SYP-F | TGGGGACTACTCCTCGTCAG |
| SYP-R | CACATGAAGGCGAACACAGC |
| NCAM1-F | AACCCAGTGCACCTAAGCTC |
| NCAM1-R | CTGGTTTCCACTCGGAGGAG |
| CD44-F | CAGCTCATACCAGCCATCCA |
| CD44-R | TGGGGTGTGAGATTGGGTTG |
| NCAPG-F | TGAACGTTCGAGCGGTAAAT |
| NCAPG-R | CCTCCTTAATCGACAGCAGC |
| WNT5A-F | TCCTCTCGCCCATGGAATTA |
| WNT5A -R | CATTGCACTTCCAGCCATCC |
| POLA1-F | ATTCGAAGCTGGTTCAGGCA |
| POLA1-R | TCGGCCATCTTCCACATAGC |
| GAPDH-F | AGAAGGCTGGGGCTCATTTG |
| GAPDH -R | GCAGGAGGCATTGCTGATGAT |
| U6-F | CGCTTCGGCAGCACATATAC |
| U6-R | TTCACGAATTTGCGTGTCATC |
| miR-1296-5p-F | CGTTAGGGCCCTGGCTCC |
| miR-1296-5p-RT | GTCGTATCCAGTGCAGGGTCCGAGGTATTCGCACTGGATACGACGGAGAT |
| miR-326-F | TCAGCCTCTGGGCCCTTC |
| miR-326-RT | GTCGTATCCAGTGCAGGGTCCGAGGTATTCGCACTGGATACGACCTGGAG |
| miR-1306-5p-F | GCCACCTCCCCTGCAAA |
| miR-1306-5p-RT | GTCGTATCCAGTGCAGGGTCCGAGGTATTCGCACTGGATACGACTGGACG |
| miR-1976-F | CGCCTCCTGCCCTCCT |
| miR-1976-RT | GTCGTATCCAGTGCAGGGTCCGAGGTATTCGCACTGGATACGACACAGCA |
| miR-296-5p-F | GTATAGGGCCCCCCCTCA |
| miR-296-5p-RT | GTCGTATCCAGTGCAGGGTCCGAGGTATTCGCACTGGATACGACACAGGA |
| miRNA-R | AGTGCAGGGTCCGAGGTATT |
